# Flux melting of metal–organic frameworks†
†Electronic supplementary information (ESI) available. See DOI: 10.1039/c8sc04044c


**DOI:** 10.1039/c8sc04044c

**Published:** 2019-02-12

**Authors:** Louis Longley, Sean M. Collins, Shichun Li, Glen J. Smales, Ilknur Erucar, Ang Qiao, Jingwei Hou, Cara M. Doherty,, Aaron W. Thornton, Anita J. Hill, Xiao Yu, Nicholas J. Terrill, Andrew J. Smith, Seth M. Cohen, Paul A. Midgley, David A. Keen, Shane G. Telfer, Thomas D. Bennett

**Affiliations:** a Department of Materials Science and Metallurgy , University of Cambridge , Charles Babbage Road , Cambridge , CB3 0FS , UK . Email: tdb35@cam.ac.uk; b Institute of Chemical Materials , China Academy of Engineering Physics , Mianyang 621900 , China; c Department of Chemistry , University College London , Gordon Street , London , WC1H 0AJ , UK; d Diamond Light Source Ltd , Diamond House, Harwell Science and Innovation Campus , Didcot OX11 0DE , UK; e Department of Natural and Mathematical Sciences , Faculty of Engineering , Ozyegin University , Istanbul , Turkey; f State Key Laboratory of Silicate Materials for Architectures , Wuhan University of Technology , Wuhan 430070 , China; g Future Industries , Commonwealth Scientific and Industrial Research Organisation , Clayton South , Victoria 3168 , Australia; h Department of Chemistry and Biochemistry , University of California, San Diego , La Jolla , California 92023-0358 , USA; i ISIS Facility , Rutherford Appleton Laboratory , Harwell Campus , Didcot , Oxon OX11 0QX , UK; j MacDiarmid Institute for Advanced Materials and Nanotechnology , Institute of Fundamental Sciences , Massey University , Palmerston North 4442 , New Zealand

## Abstract

Recent demonstrations of melting in the metal–organic framework (MOF) family have created interest in the interfacial domain between inorganic glasses and amorphous organic polymers. The chemical and physical behaviour of porous hybrid liquids and glasses is of particular interest, though opportunities are limited by the inaccessible melting temperatures of many MOFs. Here, we show that the processing technique of flux melting, ‘borrowed’ from the inorganic domain, may be applied in order to melt ZIF-8, a material which does not possess an accessible liquid state in the pure form. Effectively, we employ the high-temperature liquid state of one MOF as a solvent for a secondary, non-melting MOF component. Differential scanning calorimetry, small- and wide-angle X-ray scattering, electron microscopy and X-ray total scattering techniques are used to show the flux melting of the crystalline component within the liquid. Gas adsorption and positron annihilation lifetime spectroscopy measurements show that this results in enhanced, accessible porosity to a range of guest molecules in the resultant flux melted MOF glass.

## Abstract 2

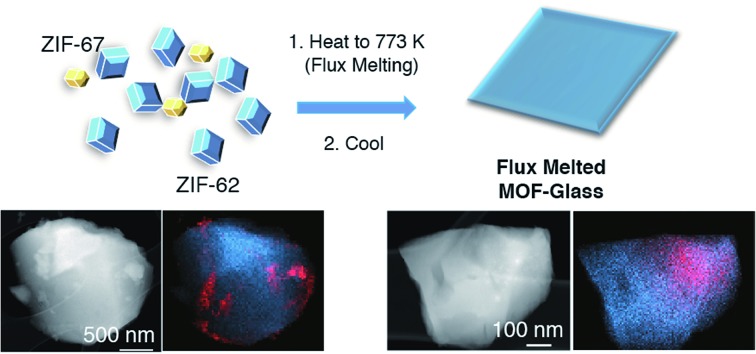
We show flux melting by using a liquid MOF as a solvent for a secondary, non-melting MOF component.

## Introduction

Porous three dimensional materials formed by the self-assembly of inorganic nodes connected by organic ligands or, as they are commonly known, metal–organic frameworks (MOFs),^1^ remain of extreme interest to the scientific community. The continuation of new materials discovery, combined with an improved understanding of the relationship between structure, property and application, drives intense research into their use in carbon capture, clean water production, catalysis, drug delivery, and light harvesting.^2^,^3^ Their thermomechanical properties have also generated a surge of recent studies, revolving around flexibility,^4^ negative gas adsorption,^5^ and defect-dependent properties.^6^ Rapid developments have also been made in asserting control over macroscale MOF architectures,^7^ such as membranes,^8^ monoliths,^9^ and thin films.^10^ Although many MOFs can be processed into pellet forms, their mechanical instabilities are not conducive to processing of their nano-crystalline structures into bulk structures which are free from grain boundaries.^11^ Set against this backdrop, there remains a necessity for MOFs in macroscale architectures that retain porous properties, but circumvent the drawbacks associated with processing and handling microcrystalline powders.

Zeolitic imidazolate frameworks (ZIFs) are a subset of MOFs in which tetrahedral metal centers are connected by imidazolate (Im, C_3_H_3_N_2_^–^) based ligands.^12^,^13^ Amongst these, the prototypical framework ZIF-8, Zn(mIm)_2_ (mIm, 2-methylimidazolate, C_4_H_5_N_2_^–^), is investigated extensively.^14^,^15^ For example, the structure, which contains pores of diameter 11.6 Å, has been shown to exhibit selectivity for the removal of Li^+^ ions from water, courtesy of the 3.4 Å limiting pore window size.^16^ The formation of mixed-matrix membranes (MMMs) through dispersal of ZIF-8 within an organic polymer has also been attempted,^17^ though products may suffer from poor adhesion between the two components. Cross-linking of the ZIF to the organic matrix, through either amine surface functionalization of the ZIF, or from high-temperature heat treatment of the MMM, have been investigated as potential solutions to the problem of chemical compatibility between the two components.^18^,^19^

Recently, several members of the ZIF family have been observed to melt upon heating to temperatures above 673 K.^20^ Cooling the liquid ZIF below these temperatures potentially allows the ZIF to be shaped and handled akin to a conventional silicate glass. However, the temperature window over which these materials remain intact in their liquid state is bounded by the temperature of thermal decomposition (*T*_d_),^21^ which is up to *ca.* 100 K higher than the melting temperature (*T*_m_). The [Zn(Im)_2_] glasses produced upon cooling from the liquid state possess continuous random networks, mimicking that of amorphous SiO_2_. The dominant Zn–N coordination bonding in the glass state means that they form a new, 4^th^ category of melt-quenched glasses, distinct from the inorganic (non-metallic), organic and metallic glass categories known at present.^22^ The melting behaviour of ZIFs, alongside that of phosphate-based porous coordination polymers,^23^,^24^ therefore opens up unexplored avenues in the synthesis and processing of new MOF-based glasses.^25^

The melting process in metal-imidazolate and -phosphate coordination polymer/metal–organic framework families has been observed to obey Lindemann's law,^20^,^24^,^26^ in which the ratio of the mean thermal atomic displacement of a species, and the distance to the nearest neighbour, approaches 0.1–0.13 at the melting temperature. A microscopic structural view of ZIF melting, obtained by molecular dynamics simulation, shows that Zn–Im bond breaking is a rare event. This rare event is followed by movement of the Im ligand away from the now under coordinated Zn^2+^ center, before association of a different imidazolate. This melting process, which has been likened to hydrogen bond switching in water,^20^ has only been observed in ZIFs containing the Im species. Other materials, including ZIF-8, do not melt^27^ and this places severe constrictions on the chemical and network functionality of the resultant glasses. Pathways are therefore being sought to reduce the *T*_m_ of non-melting ZIF structures below *T*_d_.

In the molten salt domain, the problem of reducing *T*_m_ is approached through use of a flux. For example, Na_2_O (*T*_m_ ≈ 1400 K) is used to lower the melting temperature of SiO_2_ (*T*_m_ ≈ 2000 K),^28^ whilst in addition molten oxide fluxes enable production of bulk metallic glasses.^29^ Organic analogies also exist, in the use of ionic liquids as solvents for secondary species.^30^,^31^ Encouraged by the similarities between inorganic glasses and those formed by melting ZIFs, we hypothesized that the high-temperature liquid state of a ZIF may serve as a flux – that is, a solvent – for other ZIFs. This strategy is successfully used to form a glass, derived from a high-temperature liquid of ZIF-62 [Zn(C_3_H_3_N_2_)_1.75_(C_7_H_5_N_2_)_0.25_] (bIm = benzimidazolate, C_7_H_5_N_2_^–^) and ZIF-8. The resultant flux melted glass displays increased porosity towards H_2_, compared with the pure MOF-glass.

## Experimental

### Synthesis

The synthesis of ZIF-62 was taken from Gustafsson *et al.*^32^ Specifically, solutions in DMF of Zn(NO_3_)_2_·6H_2_O (0.2 M), imidazole (1.5 M) and benzimidazole (0.2 M) were prepared, and mixed together in the ratio Zn : Im : bIm of 1 : 13.5 : 1.5. Solutions were heated at 403 K for 96 hours and cooled to ambient temperature. The microcrystalline product was washed three times in DMF, and dried at 373 K for 4 hours. ZIF-8 was purchased from Sigma Aldrich and used as received. A reported, steam-assisted synthesis was used for ZIF-67.^33^

The preparation of mixed samples was carried out in 0.5 g quantities. For example, 0.1 and 0.4 g of ZIF-8 and ZIF-62 were placed in a 10 mL stainless steel jar, along with 2 × 7 mm stainless steel balls. The mixture was then milled for 5 minutes (for Zn based samples) or 15 minutes (for Co based samples) in a Retsch MM400 grinder mill operating at 25 Hz (Fig. S1†). The different milling times were to accommodate the different initial particle sizes of ZIF-8, and ZIF-67 (Fig. S2†), given the larger initial particle sizes of the as-synthesized ZIF-67 phase. These crystalline mixtures were subsequently heated to 453 K for 3 hours to remove the solvent.

To form the glasses, 0.25 g of the evacuated crystalline mixture was placed in a ceramic crucible in a tube furnace, which was sealed and flushed with argon for 30 minutes. ZIF-8/ZIF-62 and ZIF-67/ZIF-62 mixtures were then heated at 10 K min^–1^ to the temperatures indicated in the main text. This was followed by natural cooling back to room temperature, still under flowing argon.

### Characterization

#### Differential scanning calorimetry

Experiments were conducted using a Netzsch STA 449 F1 instrument, in sealed platinum crucibles at a 10 K min^–1^ heating rate. To determine the *C*_p_ of the samples, both the baseline (blank) and the reference sample (sapphire) were measured. Simultaneous differential scanning calorimetry-thermogravimetric analyses were performed using a TA instruments Q-600 series differential scanning calorimeter, with the sample (*ca.* 7 mg) held in an alumina pan under a continuous flow of dry Ar gas. The data were obtained using a heating rate of 10 K min^–1^. Downscans were also conducted at 10 K min^–1^.

#### X-ray scattering

Powder diffraction data were collected with a Bruker-AXS D8 diffractometer using Cu K_α_ (*λ* = 1.540598 Å) radiation and a LynxEye position sensitive detector in Bragg–Brentano parafocusing geometry.

Combined small and wide angle X-ray scattering data were collected at the I22 beamline at the Diamond Light Source, UK (*λ* = 0.9998 Å, 12.401 keV). The SAXS detector was positioned at a distance of 9.23634 m from the sample as calibrated using a 100 nm period Si_3_N_4_ grating (Silson, UK), giving a usable *q* range of 0.0018–0.18 Å^–1^. The WAXS detector was positioned at a distance of 0.16474 m from the sample as calibrated using a standard CeO_2_ sample (NIST SRM 674b, Gaithersburg USA), giving a usable *q* range of 0.17–4.9 Å^–1^. Samples were loaded into 1.5 mm diameter borosilicate capillaries under argon inside a glovebox and sealed with Blu-tack and Para-film to prevent the ingress of air. Samples were heated using a Linkam THMS600 capillary stage (Linkam Scientific, UK) from room temperature to 873 K at 10 K min^–1^. Simultaneous SAXS/WAXS data were collected every 1 K. Data were reduced to 1D using the DAWN package^34^,^35^ and standard reduction pipelines.^36^ Values for the power law behavior of the samples were found using the power law model of SASView 4.1.1.^37^ Data were fitted over the range 0.003 ≤ *q* ≤ 0.005 Å^–1^. Particle size distributions were calculated using the McSAS package,^38^,^39^ a minimal assumption Monte Carlo method for extracting size distributions from small-angle scattering data. Data were fitted over a range of 0.002 ≤ *q* ≤ 0.18 Å^–1^ with a sphere model.

X-ray total scattering data were collected at room temperature using a PANalytical Ag-source Empyrean lab diffractometer (*λ* = 0.561 Å). Data collection was carried out using loaded 1.0 mm diameter quartz capillaries and collection times of approximately 6 h. Background, multiple scattering, container scattering, Compton scattering and absorption corrections were performed using the GudrunX program.^40^,^41^

#### Nuclear magnetic resonance spectroscopy

Solution ^1^H NMR spectra of digested samples (in a mixture of DCl (35%)/D_2_O (0.1 mL) and DMSO-d_6_ (0.5 mL)) of samples (about 6 mg) were recorded on a Bruker Avance III 400 MHz spectrometer at 293 K. Chemical shifts were referenced to the residual protio-solvent signals of DMSO-d_6_. The spectra were processed with the MestreNova Suite.

#### Electron microscopy and spectroscopy

Scanning transmission electron microscopy data were acquired using an FEI Osiris microscope equipped with a high-brightness X-FEG electron source and operated at 80 kV. The beam convergence was set to 11.0 mrad. X-ray energy dispersive spectroscopy (EDS) was acquired using a ‘Super-X’ EDS detector system with four detectors mounted symmetrically about the optic axis of the microscope (200 ms per pixel). For all spectroscopic data, images were also simultaneously recorded on annular dark field (ADF) detectors. These images contain atomic number and thickness contrast, giving information in parallel with the mapping obtained in the EDS data. Data were processed using Hyperspy,^42^ an open-source software coded in Python. EDS maps were generated by peak integration at the K_α_ X-ray emission line for each element.

#### Simulations

Full details are available in the ESI.†

#### Positron annihilation lifetime spectroscopy

^22^NaCl, which was sealed in a thin Mylar envelope, was used as the source of positrons. The samples were packed to 2 mm thickness surrounding the positron source. The *o*-Ps lifetime measurements were taken under vacuum (1 × 10^–5^ Torr) at 298 K using an EG&G Ortec spectrometer at a rate of 4.5 × 10^6^ counts per sample. The spectra were fitted to 4 lifetime components with the first two components accounting for *p*-Ps and free annihilation respectively. The 3rd and 4th component lifetimes (*τ*_3_ and *τ*_4_) were related to *o*-Ps annihilation and are correlated to the average pore sizes within the materials. The lifetimes were converted to pore sizes by using the Tao–Eldrup quantum-based formulation with a spherical pore geometry.^43^ A full description of the technique can be found in a previous study.^44^

#### Gas adsorption

Gas adsorption isotherms were measured by a volumetric method using ultra-high purity gases. Prior to analysis, the samples were degassed under a dynamic vacuum at 10^–6^ Torr for 10–20 hours at 130–250 °C. Accurate sample masses were calculated using degassed samples after sample tubes were backfilled with nitrogen. Where possible, BET surface areas were calculated from adsorption isotherms according to established procedures.^45^

## Results and discussion

### Thermal characterization of flux melting

**Fig. 1:**
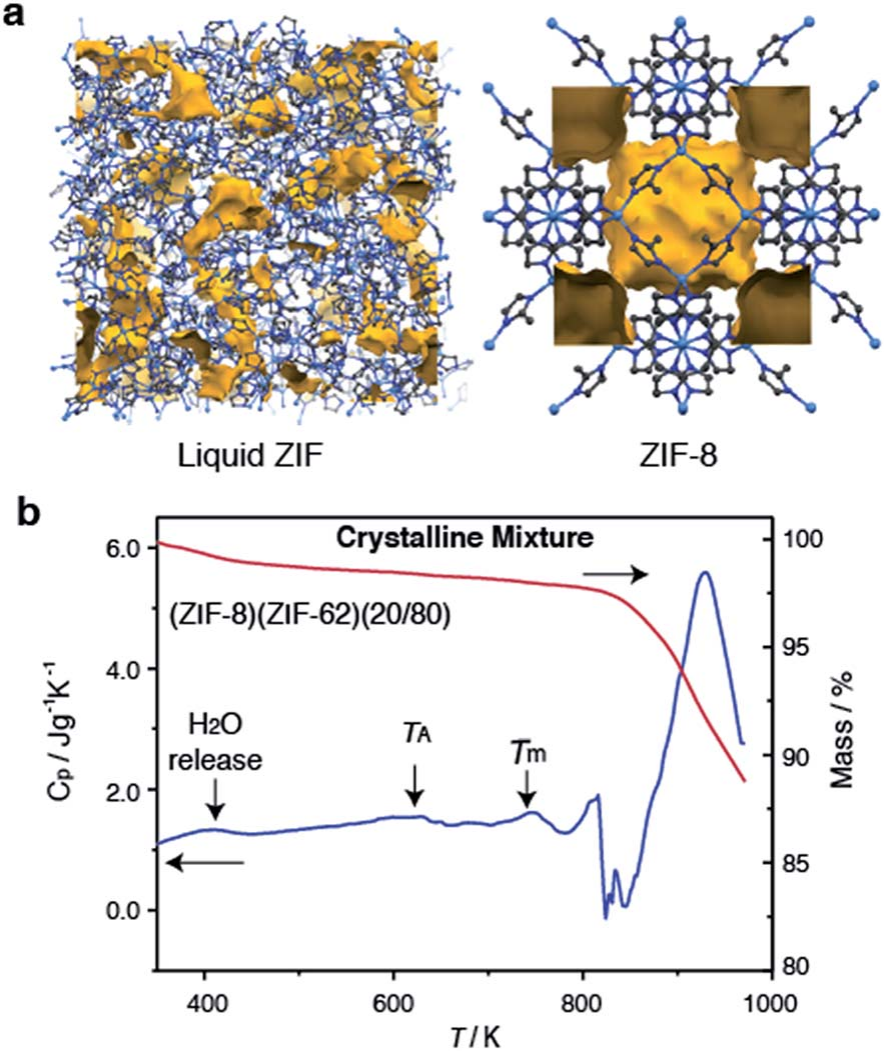
(a) Atomic configuration, *ca.* 50 Å^3^, of a high-temperature liquid ZIF obtained from a previous publication through computational, and experimental neutron and synchrotron pair distribution function modelling.^20^ Also included is the unit cell of ZIF-8.^12^ Zn – light blue, N – dark blue, C – grey, H – omitted for clarity. Void space – yellow. (b) Isobaric heat capacity (*C*_p_) and mass as a function of temperature for (ZIF-8)(ZIF-62)(20/80), at a heating rate of 10 K min^–1^.

Selection of the two components was based upon the requirement for an accessible and reasonably wide temperature region over which the liquid, and crystalline MOFs, were both stable. That is, the two components should obey the condition *T*_m1_ < *T*_d2_, where *T*_m1_ refers to the melting temperature of structure 1, the liquid-forming MOF, and *T*_d2_ to the decomposition temperature, or upper stability limit, of the crystalline form of component 2. A suitable combination was found (Fig. 1a) using: (i) the comparatively low *T*_m_ of *ca.* 710 K established for ZIF-62 [Zn(C_3_H_3_N_2_)_1.75_(C_7_H_5_N_2_)_0.25_],^46^ and (ii) the relatively high *T*_d_ of ZIF-8 (*ca.* 800 K at a heating rate of 10 K min^–1^).^21^

ZIF-62 was synthesized according to literature procedures, and combined with a sample of commercially purchased ZIF-8. Specifically, the two microcrystalline samples were mixed together in a 20/80 (ZIF-8/ZIF-62) wt/wt ratio, and ball-milled for 5 minutes to homogenize the sample. The sample was then evacuated by heating at 453 K for 3 hours. This evacuation of solvent did not result in a change in crystal structure (Fig. S1 and S2†). The resultant mix of crystalline frameworks is hereby referred to as (ZIF-8)(ZIF-62)(20/80). Differential scanning calorimetry (DSC) experiments were performed up to 973 K in an inert argon atmosphere (Fig. 1b). A broad endotherm at *ca.* 600 K indicative of thermal amorphisation, followed by an endothermic melting peak at *ca.* 730 K was observed, broadly consistent with prior observations.^46^

**Fig. 2:**
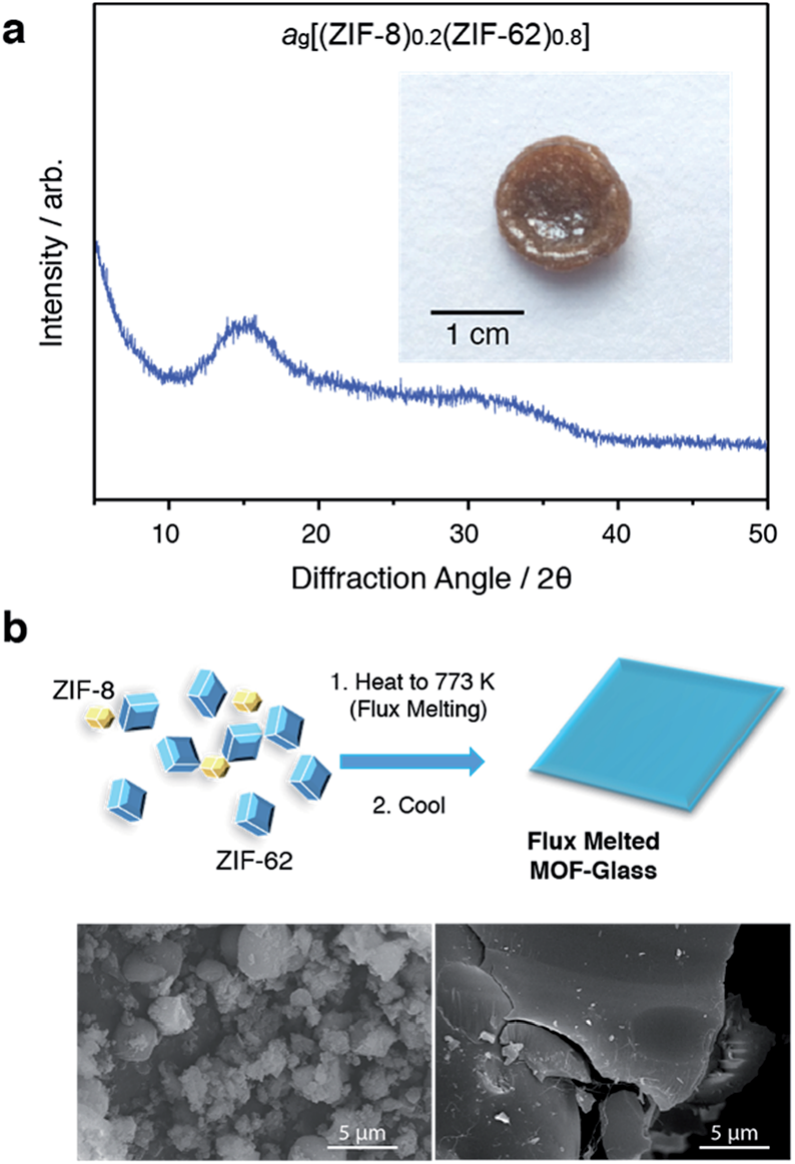
(a) Powder X-ray diffraction pattern of the glass formed after quenching from 773 K, and (inset) optical image. (b) Schematic of flux melted glass formation and SEM images of (left) (ZIF-8)(ZIF-62)(20/80) and (right) a_g_[(ZIF-8)_0.2_(ZIF-62)_0.8_].

In a separate, simultaneous differential scanning calorimetry-thermogravimetric (SDT) experiment, (ZIF-8)(ZIF-62)(20/80) was heated to 773 K at a rate of 10 K min^–1^, *i.e.* above the melting temperature of ZIF-62, and then quenched at a rate of 10 K min^–1^ back to room temperature. This produced a solid, self-supporting monolith, hereby referred to as a_g_[(ZIF-8)_0.2_(ZIF-62)_0.8_], of strikingly different external appearance to the microcrystalline mixture prior to heating (Fig. 2a inset). This terminology differentiates the flux melted glasses, from metal–organic framework blends, *e.g.* (ZIF-4)_0.5_(ZIF-62)_0.5_,^47^ in which both constituent amorphous MOF component structures appear to remain intact. Scanning electron microscopy (Fig. 2b and S2†) demonstrated that the individual particles coalesce upon their transformation into a_g_[(ZIF-8)_0.2_(ZIF-62)_0.8_], with no distinct, remnant particles from either ZIF-8 or ZIF-62 observable in this material. Consistent with these observations, the powder X-ray diffraction (PXRD) pattern of a_g_[(ZIF-8)_0.2_(ZIF-62)_0.8_] contained no Bragg scattering (Fig. 2a). A sample of pure ZIF-8 was also ball-milled for 5 minutes and heated to 773 K, then subsequently cooled to room temperature (Fig. S3†). Crystallinity was shown to remain intact.

The glassy nature of a_g_[(ZIF-8)_0.2_(ZIF-62)_0.8_] was confirmed by a second DSC heating curve (Fig. S4†), which demonstrated a glass transition of *T*_g_ = 607 K. This value is greater than that for pure ZIF-62 (*T*_g_ = 591 K).^46^ Thermogravimetric analysis (TGA) on the sample indicated that no mass was lost on heating to *ca.* 850 K (Fig. S5†). ^1^H nuclear magnetic resonance (NMR) spectroscopy on digested samples of a_g_[(ZIF-8)_0.2_(ZIF-62)_0.8_] confirmed the presence of the Im, mIm, and bIm linkers (Fig. S6†).

### Structural evolution during flux melting

**Fig. 3:**
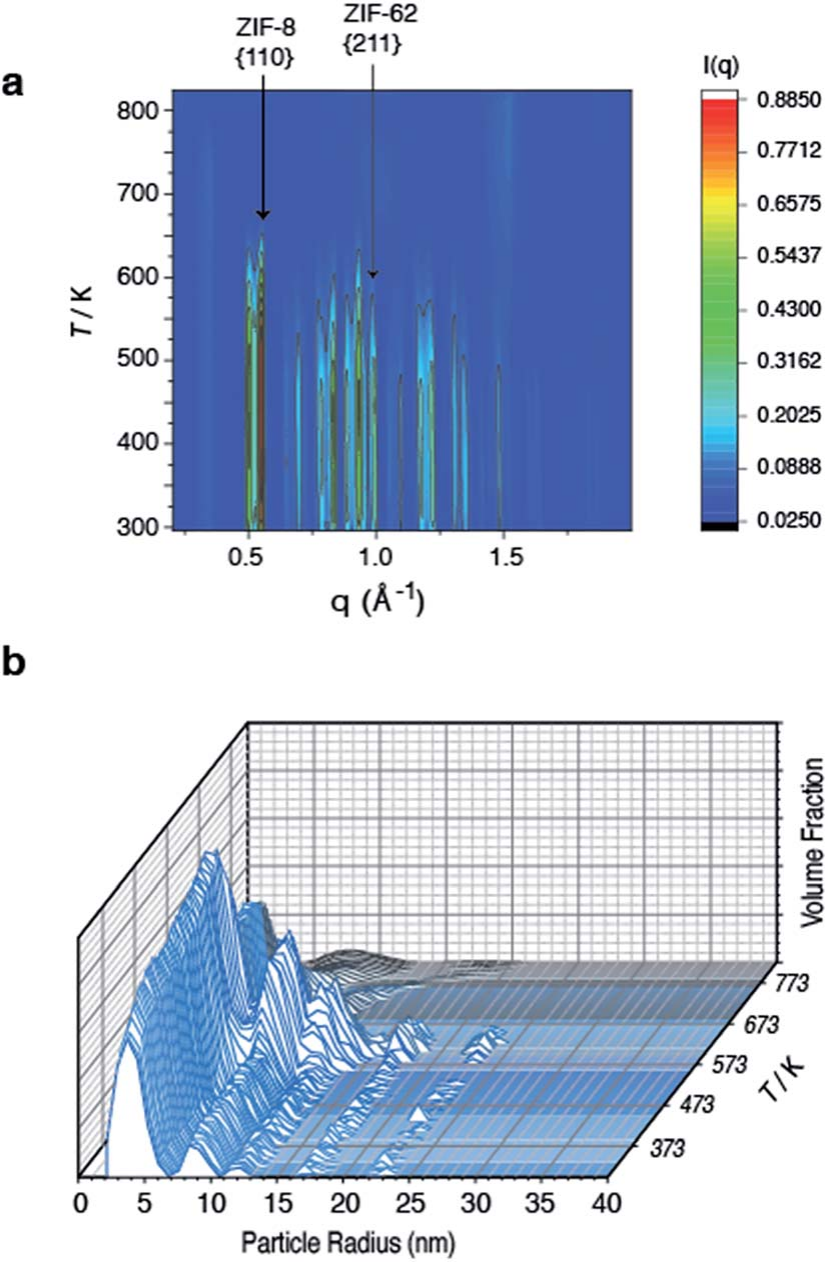
Temperature resolved diffraction of (a) WAXS profile of (ZIF-8)(ZIF-62)(20/80) and (b) volume fraction distributions of (ZIF-8)(ZIF-62)(20/80).

To further investigate the structural changes upon melting, *in situ* wide-angle X-ray scattering (WAXS) data were collected on a sample of (ZIF-8)(ZIF-62)(20/80) at the Diamond Light Source. As in prior studies, where the technique has proven useful in elucidating the mechanisms of MOF particle growth in solution,^48^ the WAXS data were combined with simultaneously collected small-angle X-ray scattering (SAXS) data. Amorphization of ZIF-62, indicated by the disappearance of the (211) peak in the temperature resolved WAXS profile, takes place at *ca.* 600 K (Fig. 3a), consistent with previous observations and the DSC trace (Fig. 1).^46^ The remaining Bragg diffraction from ZIF-8, for example the (110) peak, then disappears by *ca.* 650 K.

Porod fitting of the variable temperature SAXS profile (Fig. S7 and S8†) at room temperature, reveals that the decay in SAXS signal follows power law behavior of the form *q*^–*α*^, where *α* = 3.65. This remains constant to *ca.* 573 K, before dropping to *α* = 3.55, and then starting to rapidly increase at *ca.* 650 K. At *ca.* 750 K, the signal reaches a maximum of *α* = 4.00 and starts to decrease rapidly. This data matches the DSC data presented (Fig. S4†), where a marked increase in heat flow to the sample starts at *ca.* 650 K, which then reaches a maximum at *ca.* 750 K.

In a previous experiment, Porod fitting of variable temperature SAXS data taken on a pure sample of ZIF-62 demonstrated *α* to reach a maximum of 3.9, at *ca.* 693 K, *i.e.* in the liquid state.^47^ The maximum value of *α* = 4.00 at 750 K thus indicates loss of the internal pore structure of ZIF-8 at this temperature. Calculation of the volume weighted fraction of particle sizes below the observable limit of 310 nm diameter indicates the gradual onset of particle coalescence at *ca.* 553 K (Fig. 3b). Scattering from the original particles above 5 nm in diameter ceases at temperatures approaching 673 K, though the population of 5 nm diameter particles continues and retains some independence.

Taken together, these data indicate that ZIF-62 amorphizes at *ca.* 600 K, before beginning to melt at *ca.* 650 K, *i.e.* the same temperature region in which the Bragg peaks from ZIF-8 disappear. Thus, the implication here is that the formation of the liquid phase of ZIF-62 is causal to the flux melting of ZIF-8. The apparent offset between *T*_m,WAXS_ and *T*_m,SAXS_ is therefore ascribed to amorphization before melting, which results in the disappearance of Bragg peaks (Fig. 3a). The downturn in the value of *α* (Fig. S8†) is almost identical in temperature to the maximum of the *T*_m_ peak in the DSC (Fig. S4†).

### Flux melted glass characterization

**Fig. 4:**
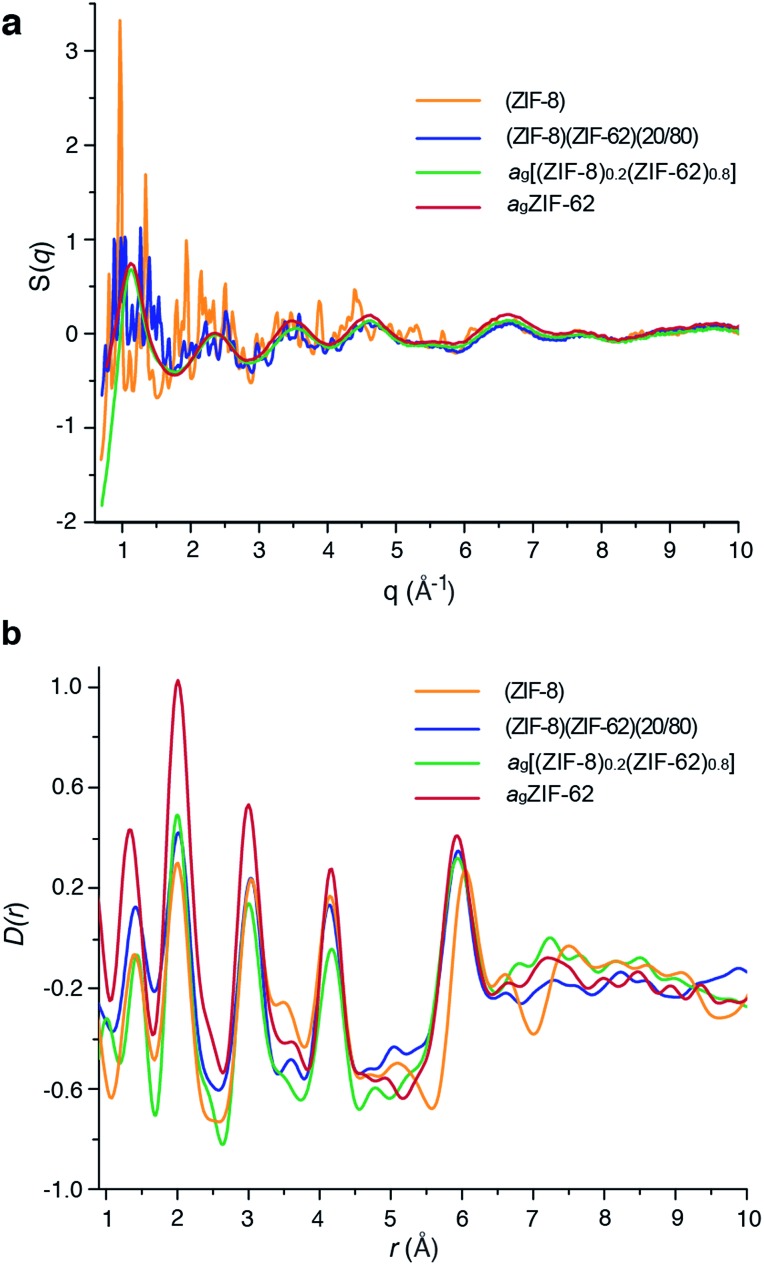
(a) Structure factors *S*(*q*) of (ZIF-8)(ZIF-62)(20/80), a_g_[(ZIF-8)_0.2_(ZIF-62)_0.8_] and ZIF-8, alongside that of a_g_ZIF-62 from a previous publication.^46^ (b) Corresponding X-ray pair distribution functions *D*(*r*).

Laboratory Ag-source total scattering experiments were carried out on crystalline ZIF-8, (ZIF-8)(ZIF-62)(20/80), and a_g_[(ZIF-8)_0.2_(ZIF-62)_0.8_] (Fig. 4a, b and S9†). The structure factor *S*(*q*) for (ZIF-8)(ZIF-62)(20/80) contained Bragg scattering, as expected for this crystalline mixture. On the other hand, consistent with its glassy nature, a_g_[(ZIF-8)_0.2_(ZIF-62)_0.8_] did not exhibit Bragg diffraction. This observation also indicated that no intact ZIF-8 crystallites remained after the melting of ZIF-62. The pair distribution functions, *D*(*r*)s, of both (ZIF-8)(ZIF-62)(20/80) and a_g_[(ZIF-8)_0.2_(ZIF-62)_0.8_], contain peaks at distances in the range 1.3–6 Å that are characteristic of ZIFs. The Zn–Zn correlation at *ca.* 6 Å in the PDFs of both (ZIF-8)(ZIF-62)(20/80) and a_g_[(ZIF-8)_0.2_(ZIF-62)_0.8_] corresponds well with a simple average of the Zn–Zn distances determined from the CIF files of ZIF-8 (6.007 Å) and ZIF-62 (5.913 Å),^12^,^49^ confirming that the short range order is maintained. The PDF of a_g_[(ZIF-8)_0.2_(ZIF-62)_0.8_] shows distinct peaks in the 6.5–8 Å region, evidencing some correlations in this region. These, through comparison with data collected previously, are ascribed to the ZIF-62 glass and not to remnant ZIF-8 crystallinity (Fig. 4b).

**Fig. 5:**
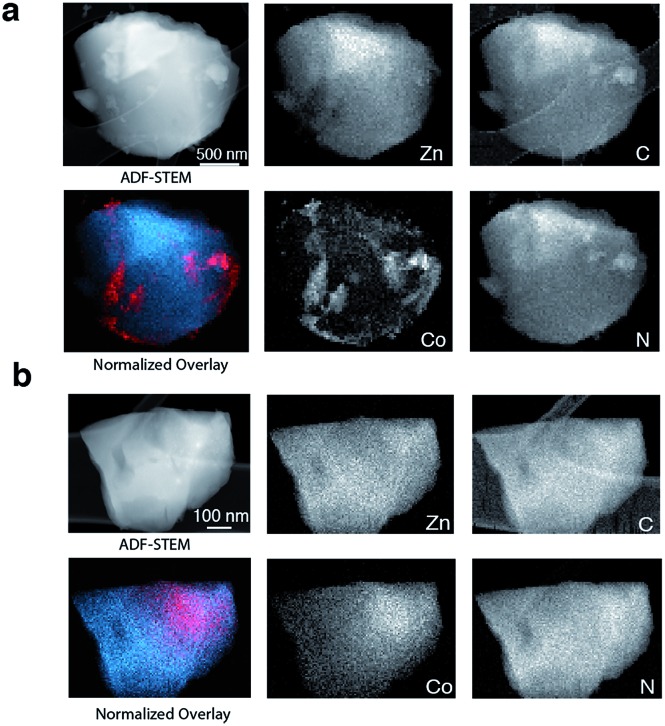
(a) ADF STEM image, EDS elemental maps for C, N, Zn and Co signals, and Zn (blue) and Co (red) component map overlay of (ZIF-67)(ZIF-62)(20/80), (b) ADF STEM image of a shard of a_g_[(ZIF-67)_0.2_(ZIF-62)_0.8_] and corresponding C, N, Zn and Co EDS elemental maps, and an overlay map of Zn (blue) and Co (red) components.

To provide chemical contrast between the two glass components, and enable the use of electron microscopy as a tool for analysing the fate of the ZIF-8 particles post-quenching, a second series of samples was prepared using ZIF-67. This framework is the isostructural cobalt(ii) analogue of ZIF-8, *i.e.* [Co(mIm)_2_]. The *T*_d_ of a pure sample of ZIF-67 was observed at *ca.* 780 K, which is below that of ZIF-8 but above the *T*_m_ of ZIF-62 (Fig. S10 and S11†).^49^ A sample of (ZIF-67)(ZIF-62)(20/80) was accordingly prepared by first synthesizing ZIF-67,^33^ and following the methodology used for the zinc based mixture. Specifically, 0.1 g of ZIF-67 was ball-milled for 15 minutes with 0.4 g ZIF-62. A sample of a_g_[(ZIF-67)_0.2_(ZIF-62)_0.8_] was then prepared by heating this mixture to 770 K in a tube furnace. Annular dark field (ADF) STEM, exhibiting thickness and atomic number contrast, and X-ray energy dispersive spectroscopy (EDS) were then used to provide chemical element maps in both the crystalline mixture and flux melted glass samples. In (ZIF-67)(ZIF-62)(20/80) (Fig. 5a), Zn, C, and N are observed in one set of particles, while Co, C and N are seen in a different, segregated set of particles.

Investigation of a shard of the flux melted glass, a_g_[(ZIF-67)_0.2_(ZIF-62)_0.8_], indicated a much more homogeneous distribution of Zn and Co (Fig. 5b). The interfaces between the two components in a_g_[(ZIF-67)_0.2_(ZIF-62)_0.8_] were also found to be much more diffuse than in (ZIF-67)(ZIF-62)(20/80), or in samples of (ZIF-67)(ZIF-62)(20/80) heated to *ca.* 100 K and *ca.* 150 K below *T*_m_ (Fig. S12–S14†). This was found to be the case across multiple particles, with elemental mapping showing similar sharp interfaces in the crystalline mixture and diffuse interfaces in the glass (Fig S15 and S16†). These maps show a two-dimensional representation of a three-dimensional interface, and therefore unambiguous analysis of individual interfaces is limited by signals arising from variation in the thickness of the particle or chemical domains within it and by uncertainty in the orientation of the interface relative to the electron beam. Here, particularly in the Co maps (Fig. 5, S15 and S16†), the preponderance of smooth interfaces observed in a_g_[(ZIF-67)_0.2_(ZIF-62)_0.8_] contrasts vividly with the prevalence of abrupt interfaces observed in (ZIF-67)(ZIF-62)(20/80).

The gradual variation of Zn and Co in a_g_[(ZIF-67)_0.2_(ZIF-62)_0.8_] suggests that zinc(ii) and cobalt(ii) are able to diffuse across significant distances in the flux-mediated melt.

### Flux melting and porosity

The permanent porosity of a pure sample of a_g_ZIF-62 was recently characterized (Table 1),^50^ with H_2_ (at 77 K) and CO_2_ (at 273 K) uptakes of 9 mL STP per g and 20 mL STP per g recorded at a pressure of 1 bar. These are lower than for the crystalline ZIF-62 framework (130 mL STP per g and 39 mL STP per g respectively). The uptake of H_2_ and CO_2_ by a_g_ZIF-62 is also lower than for a_g_ZIF-76-mbIm, which displays corresponding H_2_ and CO_2_ uptakes of *ca.* 45 mL STP per g and 35 mL STP per g.^51^ Experiments performed here also demonstrated that ZIF-62 and a_g_ZIF-62 display adsorption behaviour toward CH_4_ at 273 K (Fig. S17†).

**Table 1:** Gas adsorption properties (mL STP per g) for the crystalline and glass samples at 1 bar

Gas (kinetic diameter/Å), temperature/K	H_2_ (2.9), 77	CO_2_ (3.3), 273	O_2_ (3.46), 273	N_2_ (3.64), 77	CH_4_ (3.76), 273
ZIF-62	130^*a*^	39^*a*^	0^*a*^	0^*a*^	27
Simulated	130.5	33.9	5.2	174.2	18.6
a_g_ZIF-62	9.3^*a*^	20.1^*a*^	1.5^*a*^	0^*a*^	3.9
Simulated	40.5	7.8	0.7	45.9	1.4
(ZIF-8)(ZIF-62)(20/80)	104.5	30.7	4.4	104.3	15.2
Simulated	119.9	29.5	4.4	207.2	16.1
a_g_[(ZIF-8)_0.2_(ZIF-62)_0.8_]	27.8	18.7	1.6	1.1	4.7
Simulated	48.5	9.1	1.3	105.9	2.7

^*a*^Denotes data taken from ref. 50.

**Fig. 6:**
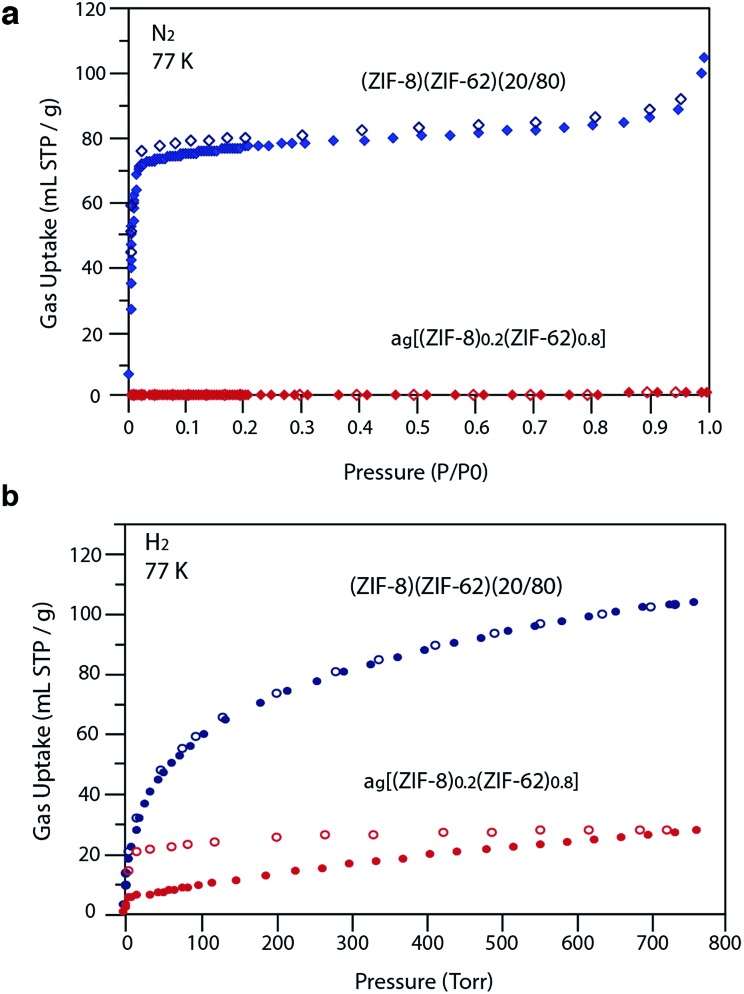
(a) N_2_ at 77 K and (b) H_2_ at 77 K gas isotherms for the zinc-based crystalline mixtures and glasses. Closed symbols represent adsorption isotherms and open symbols represent desorption isotherms.

Our observations indicate that the gas adsorption properties of (ZIF-8)(ZIF-62)(20/80) approximate a weighted average of its two components. The N_2_ adsorption isotherm of (ZIF-8)(ZIF-62)(20/80) at 77 K displays type I nitrogen behaviour, from which an accessible surface area of 350 m^2^ g^–1^ was calculated using the BET model. As the adsorbate pressure approached 1 bar, the N_2_ uptake at 77 K plateaus around 90 mL STP per g (Fig. 6a). This is consistent with the reported experimental value for N_2_ uptake in ZIF-8 of *ca.* 400 mL STP per g,^12^ and our own measurements on the material (Fig. S18†). It is in broad agreement with the 20% quantity of ZIF-8 in (ZIF-8)(ZIF-62)(20/80).

**Fig. 7:**
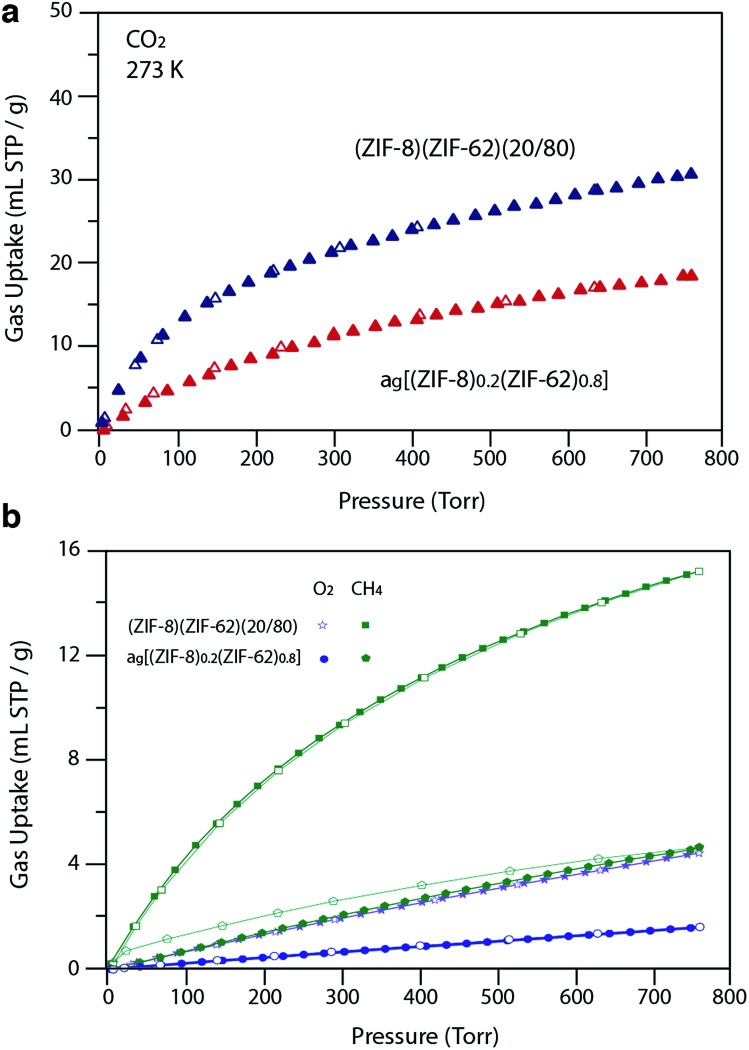
(a) CO_2_ isotherm at 273 K and (b) O_2_ and CH_4_ at 273 K isotherms for zinc based samples. Closed symbols represent adsorption isotherms and open symbols represent desorption isotherms.

For H_2_ at 77 K, (ZIF-8)(ZIF-62)(20/80) takes up 105 mL STP per g which is slightly lower than both pure ZIF-62 (130 mL STP per g) and ZIF-8 (145 mL STP per g)^12^ (Fig. 6b, Table 1). This indicates that the ball milling process used to produce (ZIF-8)(ZIF-62)(20/80) may close off a number of small pores, which are accessible to H_2_ but not larger molecules such as N_2_. (ZIF-8)(ZIF-62)(20/80) reversibly adsorbs CO_2_ at 273 K (Fig. 7a). An uptake capacity of 31 mL STP per g was observed at a pressure of 1 bar, which equates to 5.7 wt%. This uptake is only slightly lower than that of ZIF-62 (39 mL STP per g),^50^ and ZIF-8 (53 mL STP per g).^52^

A considerable degree of accessible porosity was found for a_g_[(ZIF-8)_0.2_(ZIF-62)_0.8_], *i.e.* upon flux melting and quenching of (ZIF-8)(ZIF-62)(20/80). This glass took up 19 mL STP per g of CO_2_ at 1 bar (Fig. 7a), which equates to 3.6 wt%. The desorption isotherm mapped back exactly on the adsorption isotherm, indicating the absence of significant barriers to gas diffusion. Further adsorption experiments using O_2_ and CH_4_ were also performed, which indicate that a_g_[(ZIF-8)_0.2_(ZIF-62)_0.8_] is permanently accessible to these guest molecules (Fig. 7b). The observed porosity of (ZIF-8)(ZIF-62)(20/80) towards N_2_ at 77 K is virtually eliminated by the vitrification process, however (Fig. 6a). The pore network of the ZIF-8 component, which is accessible in (ZIF-8)(ZIF-62)(20/80), appears to collapse in a_g_[(ZIF-8)_0.2_(ZIF-62)_0.8_] and places kinetic barriers to diffusion at low temperatures. This is also evident for adsorbate molecules as small as H_2_ (Fig. 6b). Here, the small amount of H_2_ uptake into a_g_[(ZIF-8)_0.2_(ZIF-62)_0.8_] confirms that it is accessible to incoming guest molecules. However, considerable hysteresis is evident in the desorption branch of this isotherm. This produced an isotherm that does not reach equilibrium between the adsorbed and free gas under any practical measurement regime.

Analysis of the CO_2_ isotherms at 273 K yielded surface areas of 325 m^2^ g^–1^ and 202 m^2^ g^–1^ for (ZIF-8)(ZIF-62)(20/80) and a_g_[(ZIF-8)_0.2_(ZIF-62)_0.8_], respectively. Pore size distributions were also calculated from these isotherms (Fig. S19 and S20†), which indicates the major pores in both materials have diameters of around 5 Å, while a smaller number of cavities have diameters centered on 9 Å. The pore volumes accessible to CO_2_ were 0.095 cm^3^ g^–1^ and 0.068 cm^3^ g^–1^, respectively.

Gas adsorption isotherms were also measured on (ZIF-67)(ZIF-62)(20/80) and a_g_[(ZIF-67)_0.2_(ZIF-62)_0.8_] (Fig. S21, Table S1†). As anticipated, the crystalline material adsorbs N_2_ at 77 K but not after vitrification. H_2_ is taken up at 77 K, but with hysteresis. On the other hand, CO_2_ adsorption at 273 K is largely preserved when the crystalline material is transformed to the glass. Surface areas of 218 m^2^ g^–1^ for (ZIF-67)(ZIF-62)(20/80) and 194 m^2^ g^–1^ for a_g_[(ZIF-67)_0.2_(ZIF-62)_0.8_] were estimated from these isotherms together with accessible pore volumes of 0.067 cm^3^ g^–1^ and 0.062 cm^3^ g^–1^, respectively. Estimated pore size distributions (Fig. S22 and S23†) paralleled their zinc counterparts. The rate of uptake of CO_2_ in these materials was measured. A comparison of these kinetics plots (Fig. S24†) reveals that the diffusion of CO_2_ in the glass is slower than in its crystalline precursor, which is consistent with its more constricted and tortuous pore network.

To provide further information on the pores present in the samples before and after vitrification, positron annihilation lifetime spectroscopy (PALS) experiments were carried out (Fig. S25 and S26, Table S2†). Measurements on crystalline ZIF-8 indicated one main cavity size of 9.5 Å diameter, which is close to the reported 11.6 Å value after accounting for van der Waals radii. A minor cavity of 23 Å was also observed in the study. (ZIF-8)(ZIF-62)(20/80) was also found to possess a major cavity with 5.8 Å diameter, alongside a secondary cavity with a diameter of 10 Å. This is consistent with the presence of a major cavity in pure ZIF-8 at 9.5 Å, and one in ZIF-4, which is closely related to ZIF-62, at 6.2 Å.^44^ PALS measurements on a_g_[(ZIF-8)_0.2_(ZIF-62)_0.8_] also show a bimodal distribution, with a similar cavity size distribution to a pure sample of a_g_ZIF-62, found previously (Fig. S25, Table S2†).^25^ The key difference between the two samples is that the smaller pore limiting aperture is larger for the a_g_[(ZIF-8)_0.2_(ZIF-62)_0.8_] glass at 3.1 Å compared to 2.5 Å for a_g_(ZIF-62). In all cases, these results are broadly consistent with the pore size distributions obtained from CO_2_ sorption analysis.

Grand canonical Monte Carlo (GCMC) simulations of gas adsorption by (ZIF-8)(ZIF-62)(20/80), for which structural models of each of the crystalline phases were derived from single-crystal X-ray diffraction, led to broad agreement between calculated and experimental data (Table 1). For example, CO_2_ uptake at 273 K in (ZIF-8)(ZIF-62)(20/80) was predicted as 30 mL STP per g, which is very close to the experimental CO_2_ measurement (31 mL STP per g). Similarly, the simulated CH_4_ uptake at 273 K of 16 mL STP per g agrees well with the experimental value of 15 mL STP per g. Simulations overestimated N_2_ adsorption at low temperature (77 K) in (ZIF-8)(ZIF-62)(20/80), where diffusion limitations prevented the ingress of the adsorbate in experimental isotherms. The source of error was proven by simulating N_2_ uptake in (ZIF-8)(ZIF-62)(20/80) at 195 K (25 mL STP per g), which agreed well with an observed experimental value at 195 K of 23 mL STP per g (ESI Table S5†).

The modelling of amorphous structures is extremely challenging due to the complexity of constructing accurate models. To provide a qualitative estimate of the gas sorption behaviour of a_g_[(ZIF-8)_0.2_(ZIF-62)_0.8_], we followed existing literature^20^,^53^ and used a molecular dynamics (MD) method to develop a model for a_g_ZIF-62. Initial configurations of ZIF-62 were melted in the NPT ensemble at 1 bar by heating to 1500 K at a rate of 100 K ps^–1^ from 300 K before quenching to 300 K at a controlled rate. Calculations of the gas adsorption behaviour of this model were then combined with those using a crystalline model of ZIF-8 (ESI†). Simulated O_2_ uptake (1 mL STP per g) at 273 K was in agreement with negligible experimental uptake of 2 mL STP per g for a_g_[(ZIF-8)_0.2_(ZIF-62)_0.8_], whilst the low predictions for CH_4_ adsorption at 273 K, agreed broadly with experimental data. Simulations overestimated N_2_ adsorption at low temperature (77 K) in a_g_[(ZIF-8)_0.2_(ZIF-62)_0.8_], though the simulated N_2_ uptake in a_g_[(ZIF-8)_0.2_(ZIF-62)_0.8_]at 273 K of 1 mL STP per g agrees well with the experimental value of 1 mL STP per g (ESI Table S5†). The over prediction of H_2_ uptake at 77 K (49 mL STP per g) compared to the experimental value (28 mL STP per g) is consistent with our assertion that the ZIF-8 structure does not remain intact within the flux melted glass (Table 1). Full details of molecular simulations are given in the ESI,† though two different configurations (imidazolate and benzimidazolate linkers with partial occupancies of 62.5% and 37.5%, respectively) of ZIF-62 were considered in molecular simulations due to the disorder in the framework. The average of the predictions agreed well with the experimental data (Table 1). Secondly, our computational approach represents the first instance where accurate predictions for the gas adsorption performances of ZIF–ZIF crystalline mixture absorbents and ZIF–ZIF glassy flux melts have been made.

## Conclusions

These results show that the concept of flux melting, that is, the use of a molten salt as a solvent, may be applicable to MOF chemistry. The flux melted glass reported here is different from the intriguing mixed matrix membrane created by Kertik *et al.*, by thermally amorphizing a ZIF-8 loaded imide polymer.^19^ This *in situ* amorphization, by heating the MMM at 623 K for up to 24 hours, was observed to cross-link ZIF-8 particles with the imide. This resulted in retention of the porous interior of the ZIF-8 component, though in an amorphous material. Here, the highly porous ZIF-8 interior does not appear to be retained, suggesting a different process to the cross-linking in the thermally treated MMM.

From a fundamental view, the successful realisation of flux melting, which uses the liquid state of ZIF-62 to facilitate the melting of ZIF-8, presents a method by which the *T*_m_ of a non-melting framework can be accessed. Use of elemental contrast in the electron microscopy experiments shows that melting of the cobalt analogue of ZIF-8 occurs quickly upon formation of the liquid ZIF-62, and results in regions of the glass which contain higher concentrations of the cobalt-containing component than others. The flux melted glass contains short range ordering reminiscent of the crystalline ZIF-62 parent phase, and a continuous random network akin to that of amorphous SiO_2_, though with accessible porosity. The increased porosity relative to the pure ZIF-62 glass is ascribed to the ZIF-8 component disrupting the close packing of the ZIF-62 matrix in the liquid phase, rather than any retention of the nanopores belonging to crystalline ZIF-8. The demonstration of porosity towards H_2_ and CO_2_, in the flux melted samples is notable, and suggests possibilities in, for example, free-standing membrane manufacture.

## Conflicts of interest

There are no conflicts to declare.

## Supplementary Material

Supplementary information
