# Supplementary material for: Flux melting of metal–organic frameworks
Source: Chem Sci. 2019 Feb 12;10(12):3592–601. doi: 10.1039/c8sc04044c (PMC6430010; doi:10.1039/c8sc04044c)
Supplement: Supplementary file 1 [file SC-010-C8SC04044C-s001.pdf]

Electronic Supplementary Material (ESI) for Chemical Science.
This journal is © The Royal Society of Chemistry 2019

Supplementary Information

Flux Melting of Metal-Organic Frameworks

Louis Longley,[a] Sean M. Collins,[a] Shichun Li,[a,b] Glen J. Smales,[c,d] Ilknur Erucar,[e] Ang Qiao,[f] Jingwei Hou,[a] Cara M. Doherty,[g] Aaron W. Thornton,[g] Anita J. Hill,[g] Xiao Yu,[h] Nicholas J. Terrill,[d] Andrew. J. Smith,[d] Seth M. Cohen,[h] Paul A. Midgley,[a] David A. Keen,[i] Shane G. Telfer [j] and Thomas D. Bennett[a]*

[a]*Department of Materials Science and Metallurgy, University of Cambridge, Charles Babbage Road, Cambridge, CB3 0FS, UK*

[b]*Institute of Chemical Materials, China Academy of Engineering Physics, Mianyang 621900, China*

[c]*Department of Chemistry, University College London, Gordon Street, London, WC1H 0AJ, UK*

[d]*Diamond Light Source Ltd, Diamond House, Harwell Science and Innovation Campus, Didcot OX11 0DE, UK*

[e]*Department of Natural and Mathematical Sciences, Faculty of Engineering, Ozyegin University, Istanbul, Turkey*

[f]*State Key Laboratory of Silicate Materials for Architectures, Wuhan University of Technology, Wuhan 430070, China*

[g]*Future Industries, Commonwealth Scientific and Industrial Research Organisation, Clayton South, Victoria 3168, Australia*

[h]*Department of Chemistry and Biochemistry, University of California, San Diego, La Jolla, California 92023-0358, United States*

[i]*ISIS Facility, Rutherford Appleton Laboratory, Harwell Campus, Didcot, Oxon OX11 0QX, UK*

[j]*MacDiarmid Institute for Advanced Materials and Nanotechnology, Institute of Fundamental Sciences, Massey University, Palmerston North 4442, New Zealand*

Email: tdb35@cam.ac.uk

Table of Contents

1. **Supplementary characterisation data** **Page 3**
2. **Supplementary gas adsorption isotherms and analysis** **Page 16**
3. **Simulated gas adsorption isotherms** **Page 21**
4. **Supplementary references** **Page 25**

1. Supplementary characterisation data

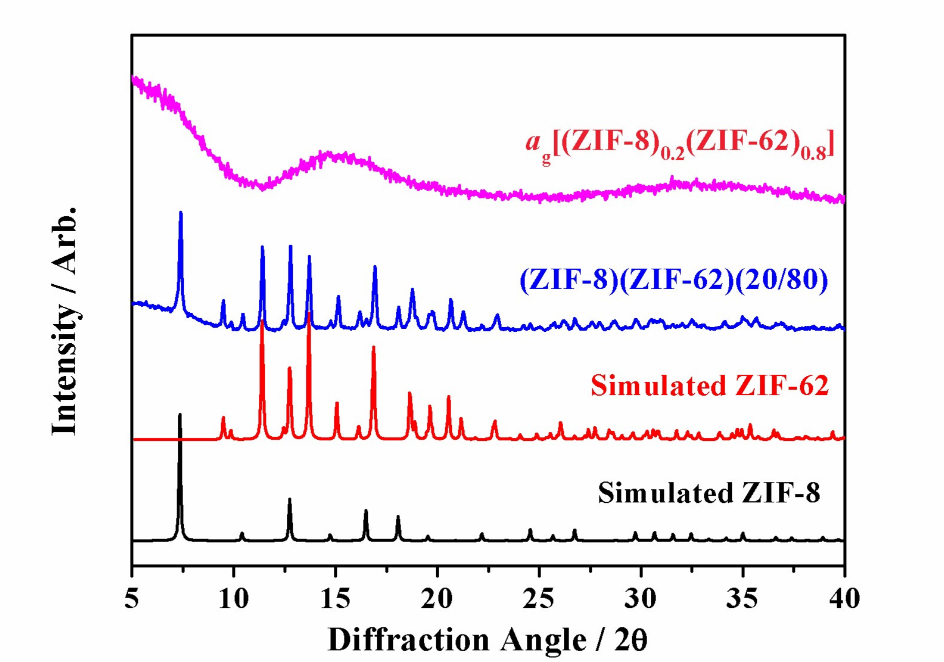

Figure S1. X-ray powder diffraction patterns of Zn-based samples. Simulated patterns for ZIF-8 and ZIF-62,[1, 2] alongside experimental patterns for (ZIF-8)(ZIF-62)(20/80) and a_g[(ZIF-8)$_{0.2}$(ZIF-62)$_{0.8}$].

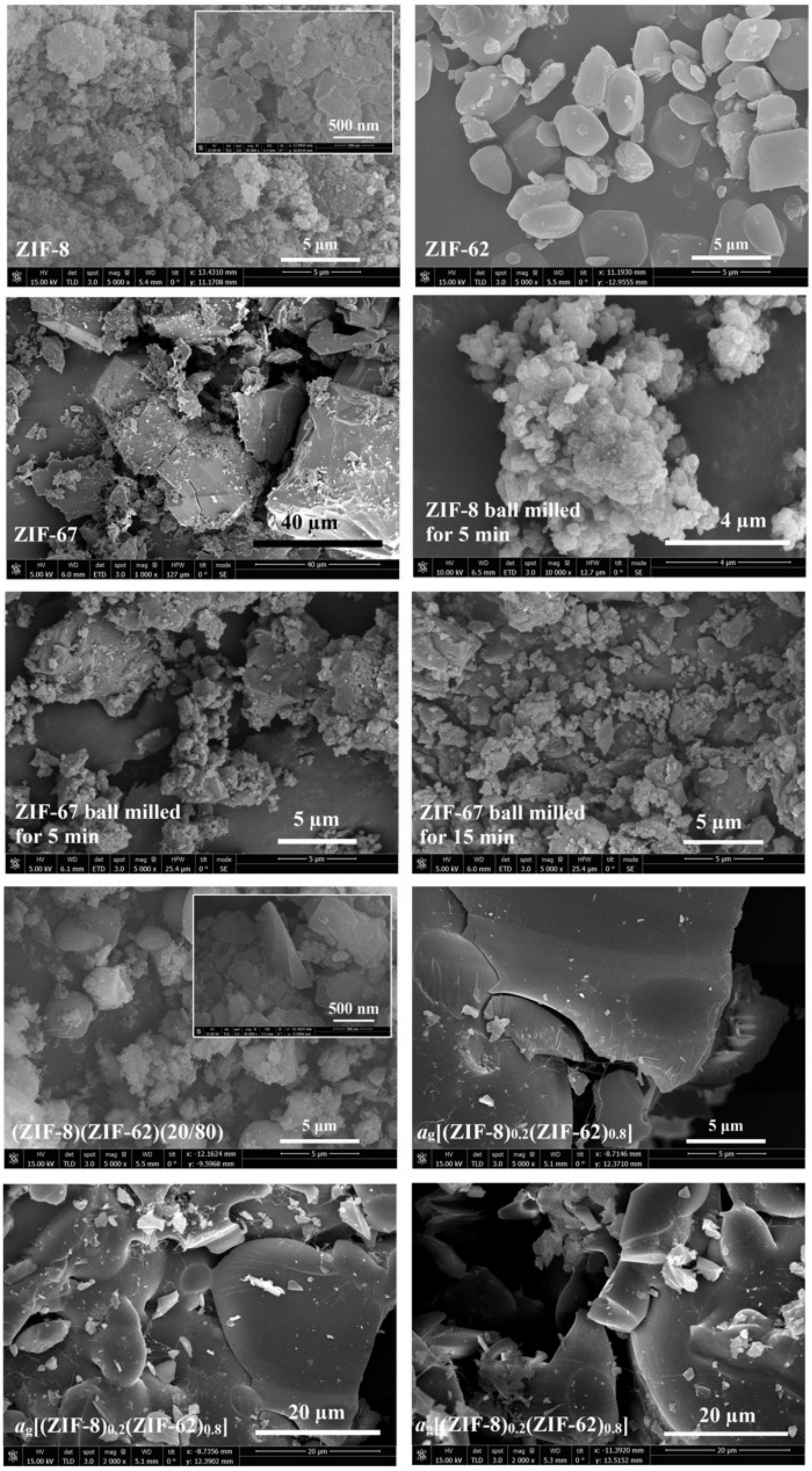

Figure S2. Scanning electron microscopy images. SEM images of ZIF-8, ZIF-62 as-synthesized, ZIF-67 as-synthesized, ZIF-8 (~500 mg) ball-milled for 5 minutes, ZIF-67 (~100 mg) ball-milled for 5 minutes, ZIF-67 (~40 mg) ball-milled for 15 minutes, the crystalline mixture of ZIF-8 and ZIF-62, i.e. (ZIF-8)(ZIF-62)(20/80), and the glass a_g[(ZIF-8)$_{0.2}$(ZIF-62)$_{0.8}$]. Amounts in brackets reflect the approximate amounts of the controls that were ball milled.

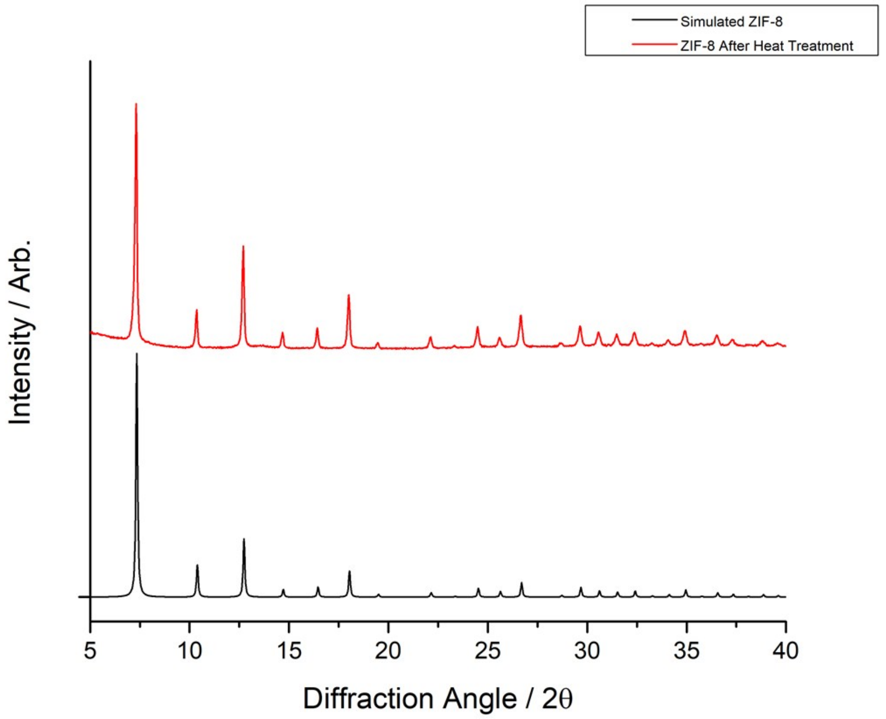

Figure S3. Powder diffraction of samples after heat treatment. Simulated[1] and experimental traces of ZIF-8. The latter was taken from a sample of ZIF-8 that was ball-milled for 5 minutes, heated to 773 K and then quenched back to room temperature at a rate of 10 K/min in an argon atmosphere.

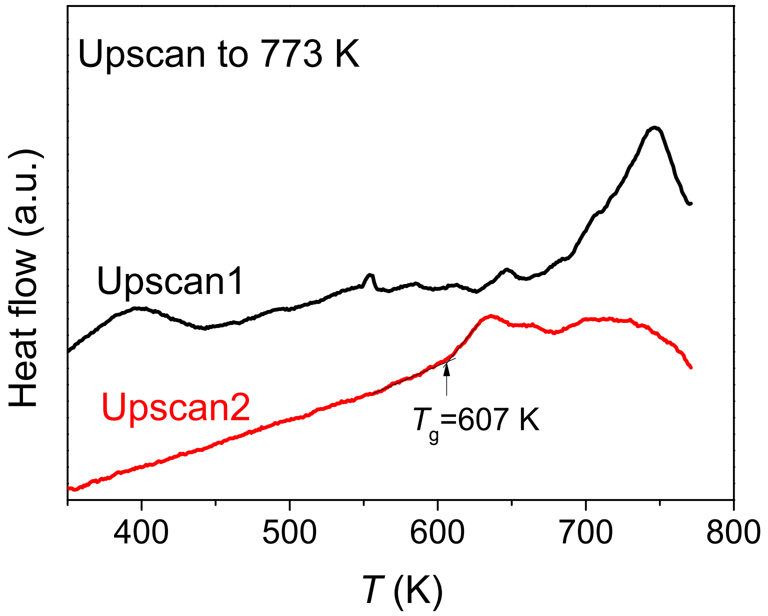

Figure S4. Glass transition identification. DSC experiments performed to 773 K on (ZIF-8)(ZIF-62)(20/80) (black), and a_g[(ZIF-8)$_{0.2}$(ZIF-62)$_{0.8}$] (red). Heating rates are 10 K/min, and the experiment was conducted in an argon atmosphere.

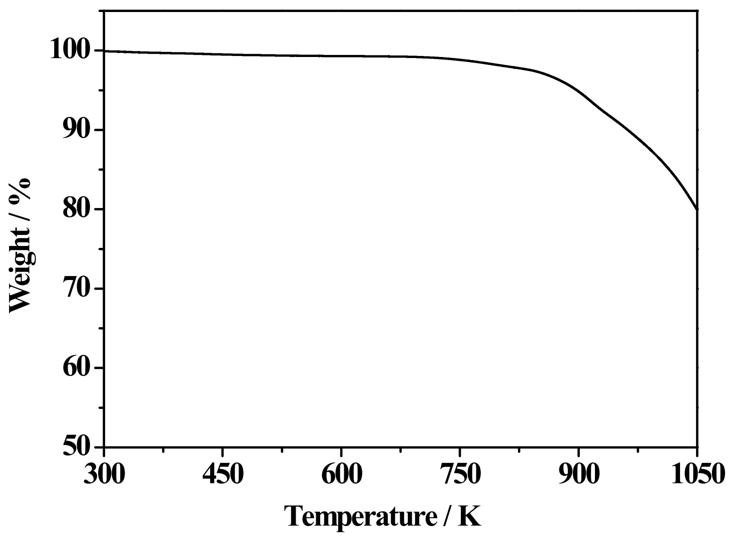

Figure S5. Thermal gravimetric analysis of a_g[(ZIF-8)$_{0.2}$(ZIF-62)$_{0.8}$]. Experiment performed at a heating rate of 10 K/min, in an argon atmosphere.

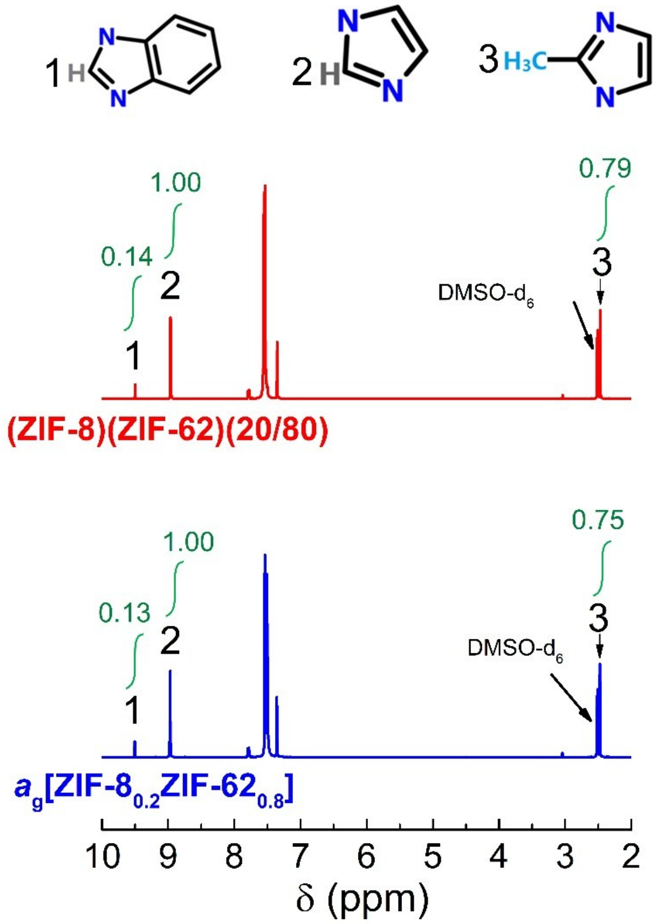

Figure S6. ^{1}H Nuclear magnetic resonance spectroscopy. ^{1}H NMR spectra of the mixture of ZIF-8 and ZIF-62, i.e. (ZIF-8)(ZIF-62)(20/80), and the glass, a_g[(ZIF-8)$_{0.2}$(ZIF-62)$_{0.8}$].

(ZIF-8)(ZIF-62)(20/80): ^{1}H NMR (400 MHz, DMSO-d_6): 2.51 (DMSO-d_6), 7.53 (HCl/H_2O), 9.0 (NC***H***N_{Im}), 9.5 (NC***H***N_{bIm}) 2.47 (NC(C***H***$_3$)N_{mIm}).

a_g[(ZIF-8)$_{0.2}$(ZIF-62)$_{0.8}$]: ^{1}H NMR (400 MHz, DMSO-d_6): 2.51 (DMSO-d_6), 7.53 (HCl/H_2O), 8.96 (NC***H***N_{Im}), 9.5 (NC***H***N_{bIm}) 2.47 (NC(C***H***$_3$)N_{mIm}).

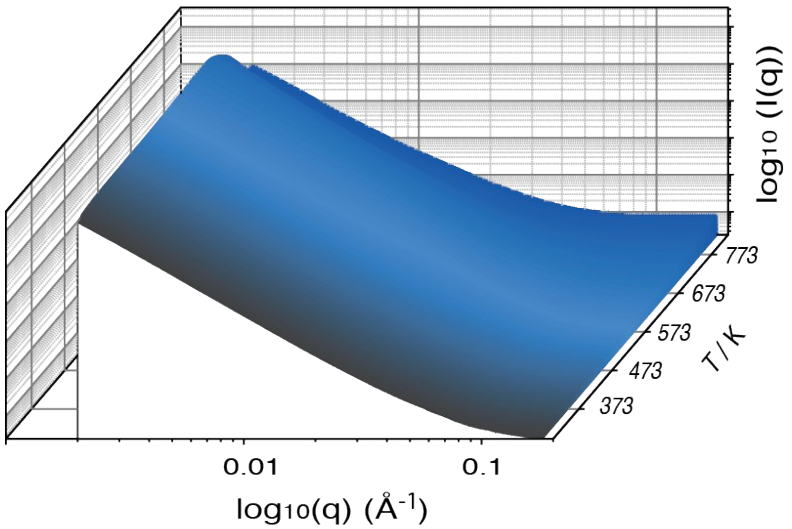

Figure S7. Temperature resolved SAXS profile of (ZIF-8)(ZIF-62)(20/80). Colour gradient included to improve readability (as a temperature indicator), and does not signify intensity.

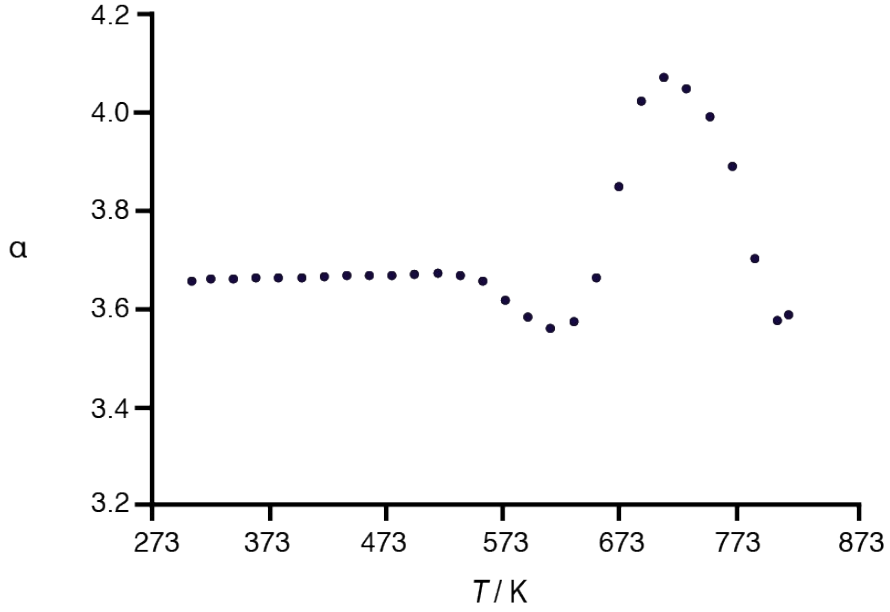

Figure S8. Porod fitting of small-angle X-ray scattering data. (ZIF-8)(ZIF-62)(20/80) variable temperature SAXS data.

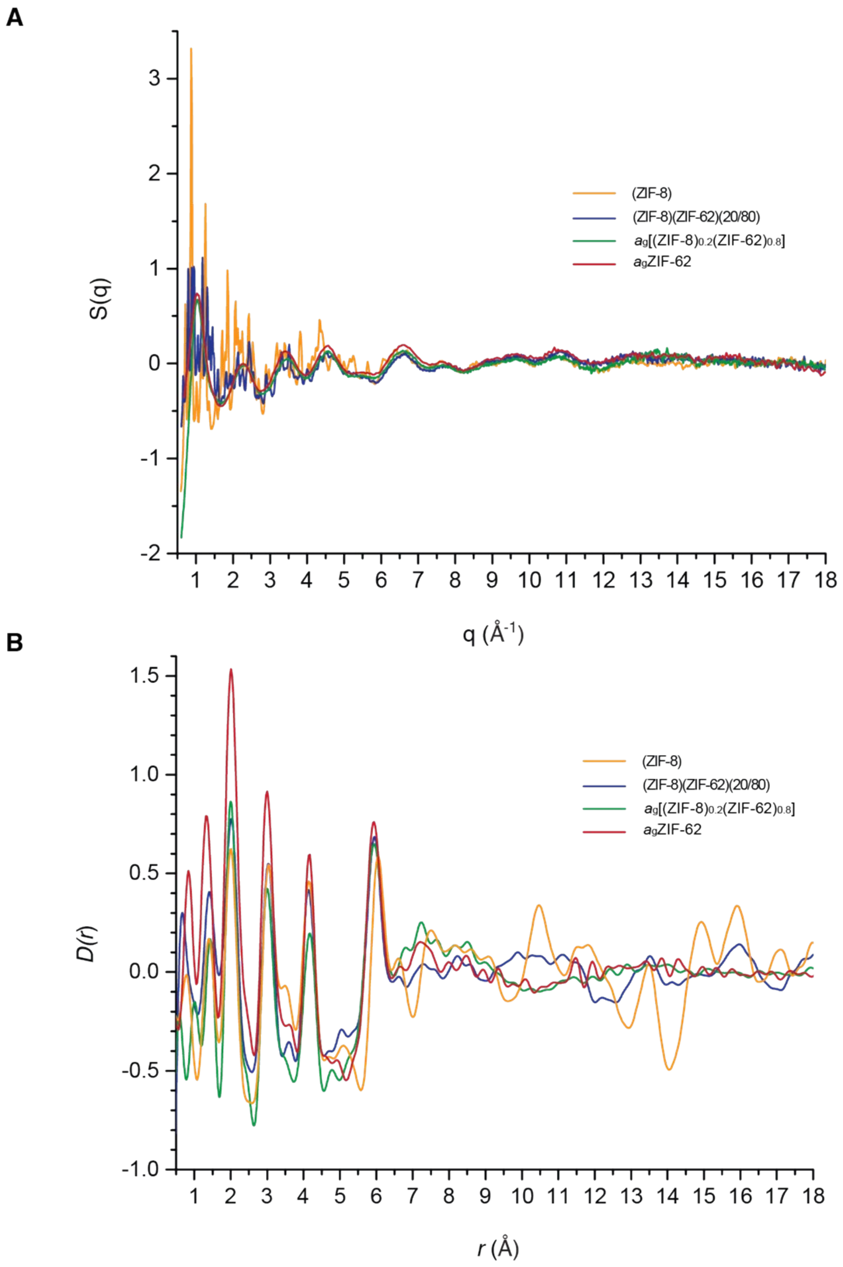

Figure S9. Total X-ray scattering data. Structure factors, *S*(q), of (ZIF-8)(ZIF-62)(20/80), a_g[(ZIF-8)$_{0.2}$(ZIF-62)$_{0.8}$], ZIF-8, and a_gZIF-62 from a prior study[3]. (d) Corresponding X-ray pair distribution functions *D*(r).

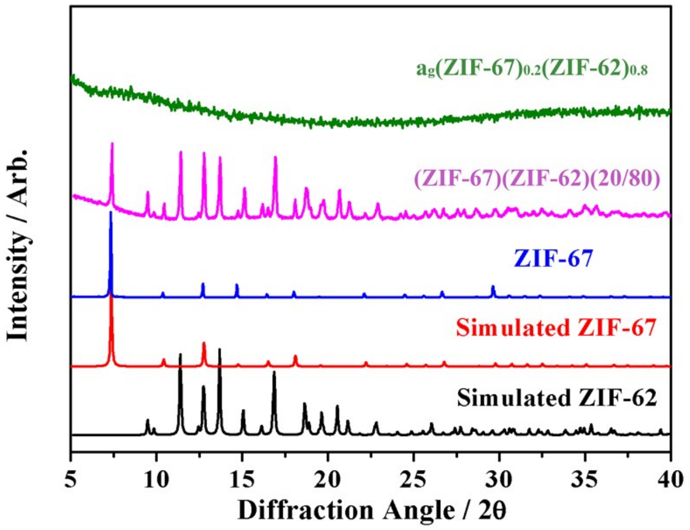

Figure S10. X-ray powder diffraction patterns of ZIF-67, (ZIF-67)(ZIF-62)(20/80) and a_g[(ZIF-67)$_{0.2}$(ZIF-62)$_{0.8}$]. Simulated ZIF-67 and ZIF-62 patterns, alongside experimental patterns for as-synthesized ZIF-67, (ZIF-67)(ZIF-62)(20/80), and the glass a_g[(ZIF-67)$_{0.2}$(ZIF-62)$_{0.8}$].

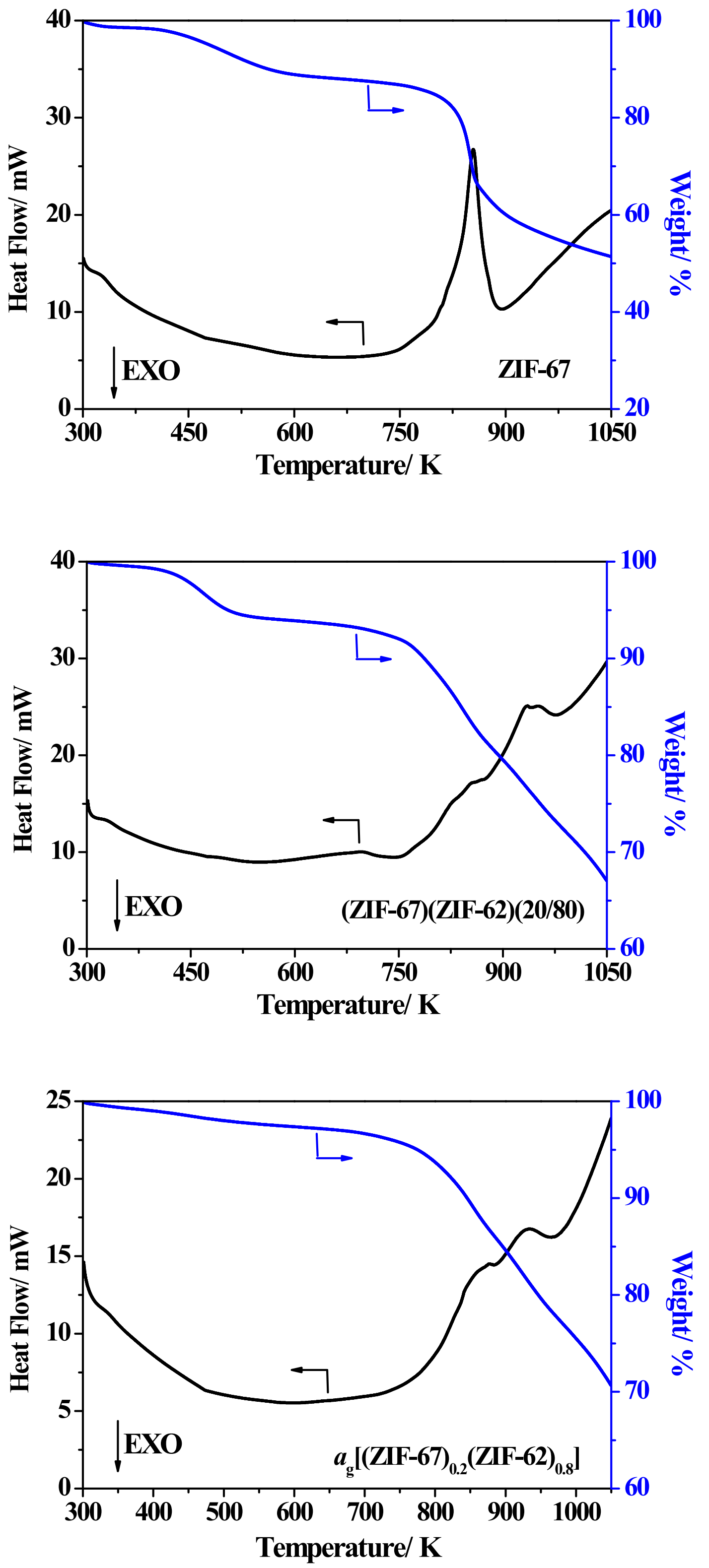

Figure S11. Differential scanning calorimetry. Experiments conducted to temperatures up to 1050 K at a rate of 10 K/min in an argon atmosphere for ZIF-67, the mixture of ZIF-67 and ZIF-62, i.e. (ZIF-67)(ZIF-62)(20/80), and the glass [a_g(ZIF-67)$_{0.2}$(ZIF-62)$_{0.8}$].

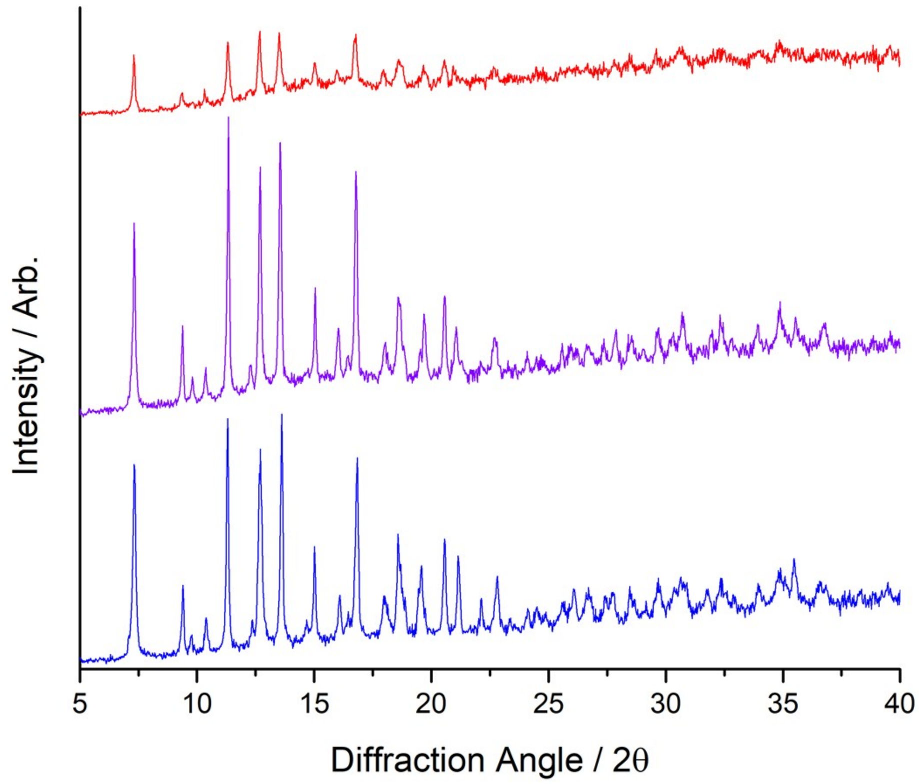

Figure S12. X-ray powder diffraction patterns of (ZIF-67)(ZIF-62)(20/80) (blue, bottom trace), (ZIF-67)(ZIF-62)(20/80) heated to 622 K (purple, middle trace), and (ZIF-67)(ZIF-62)(20/80) heated to 667 K (red, top trace). Heat treatments were conducted in a Q-600 series differential scanning calorimeter under argon at a heating rate 10 K/min.

Note on the analysis of interface characteristics from two-dimensional STEM-EDS:

The line profiles presented in Figure S13 and Figure S14 are illustrative examples of the prevailing interface characteristics recorded in the Co maps of the crystalline mixture and in the glass. Individual interface examples might be selected from each sample which do not follow these behaviours exactly. The Co maps in the main text (Figure 5) and in Figure S15 and Figure S16 depict the major characteristics of multiple interfaces across several particles. The line profiles provide additional alternative visualization of these characteristics, removing differences in image size in the presentation of the respective maps. These maps, in addition to the illustrative line profiles, show the predominance of sharp interfaces in the crystalline mixture and diffuse interfaces in the glass. Two-dimensional analysis of interfaces in electron microscopy and EDS, in general, requires an 'edge-on' interface for unambiguous analysis. As such, particles at various orientations like those encountered in the crystalline mixture and the glass are not ideal examples for individual interface analysis. Yet they still allow for convincing assessment of the most common interface properties across the entire sample. The direct measurement of individual interface characteristics may be better understood by three-dimensional characterization, and work is ongoing to establish the necessary methods to pursue this challenging question further.

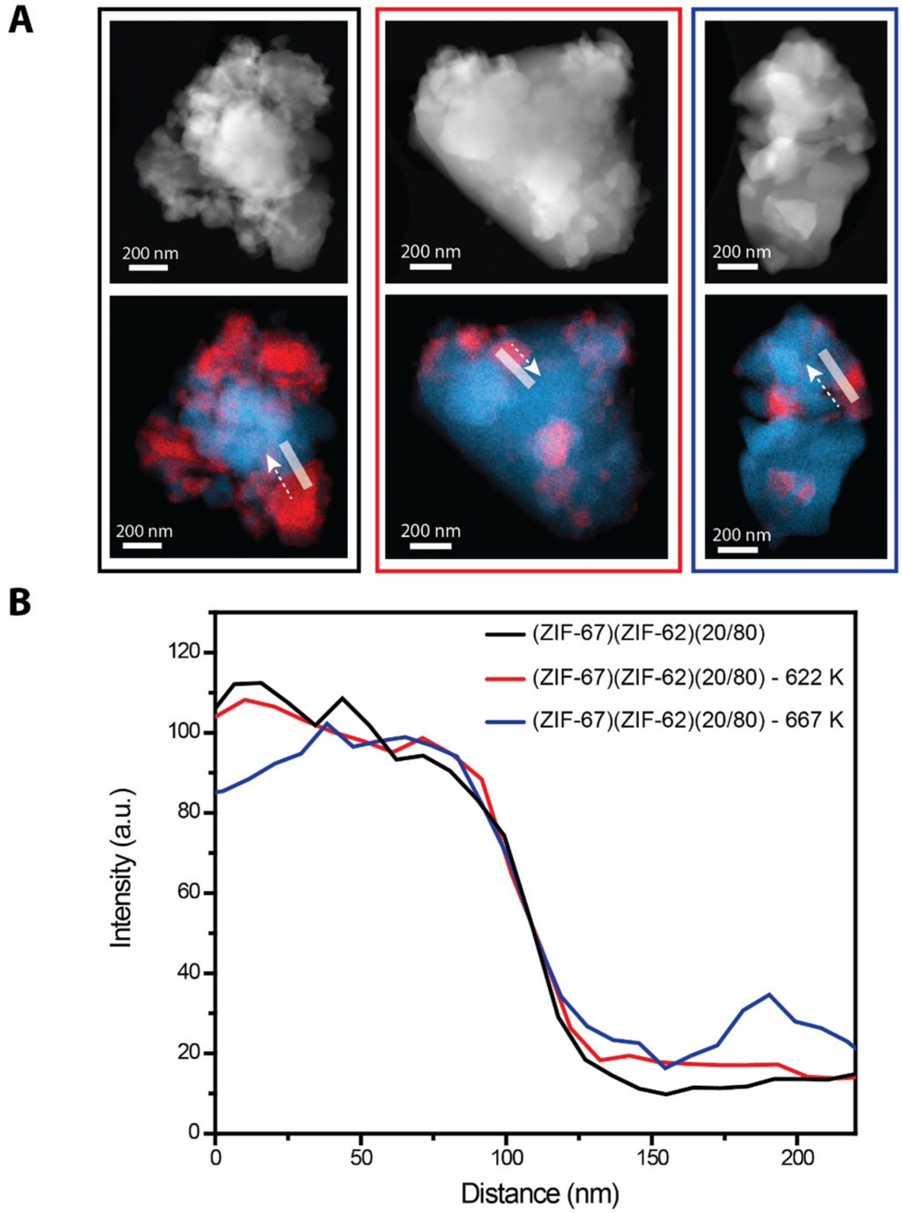

Figure S13. Line profiles of the Co signal across Co/Zn interfaces in the EDS micrographs corresponding to samples heated, in a TA instruments Q-600 series differential scanning calorimeter under argon at a heating rate 10 K/min, to temperatures below the melting point of ZIF-62. (a) ADF-STEM micrographs are shown for three conditions: as prepared, heated to 622 K, and heated to 667 K. The line profiles are marked on the EDS maps with a white line. The arrow marks the direction of the line profile. (b) The extracted line profiles for the Co intensity. There is a sharp decay of the Co intensity within <50 nm of the interface for all cases, in marked contrast with more diffuse interfaces only observed for samples heated above the melting point of ZIF-62.

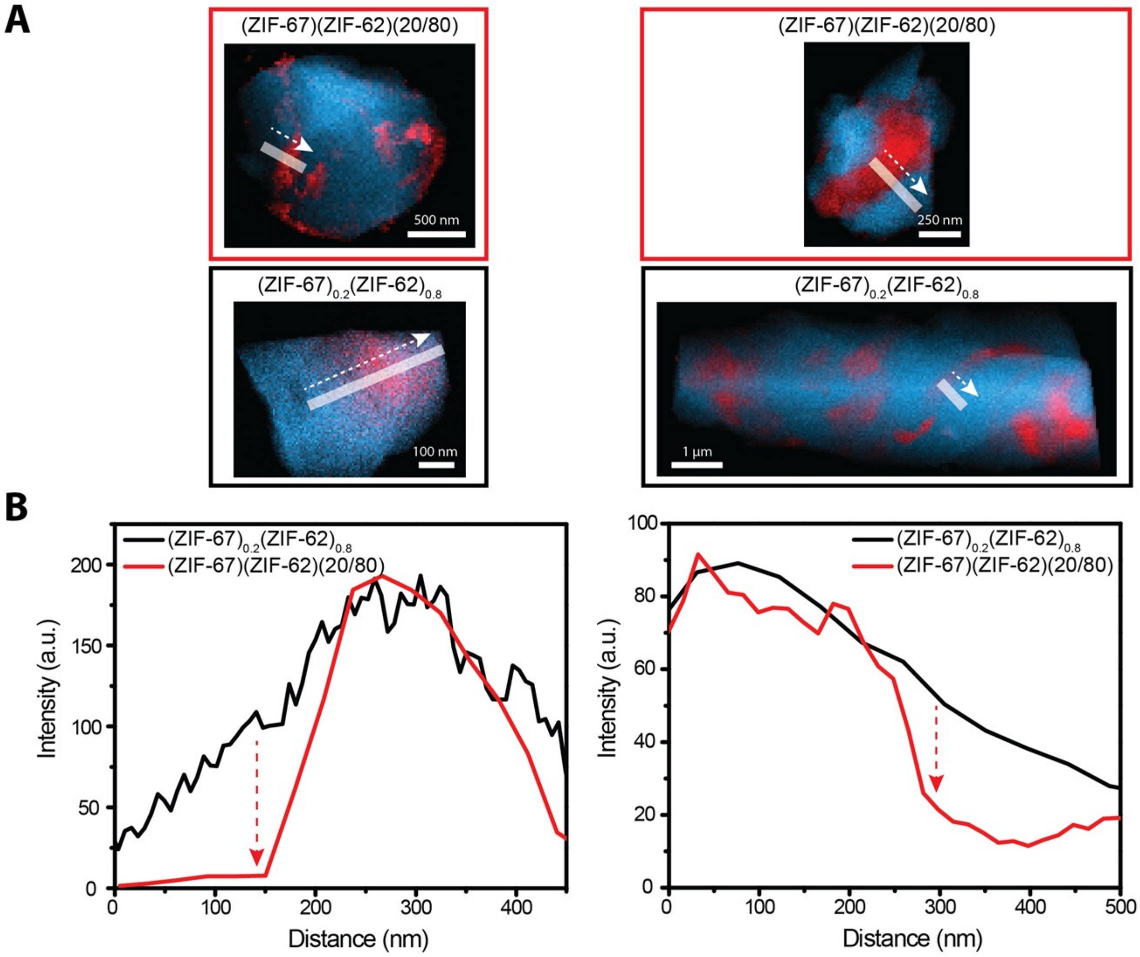

Figure S14. Figure S12. Line profiles of the Co signal across Co/Zn interfaces in the EDS micrographs presented in (Left) Figure 5 and (Right) Figures S15 and S16. (a) The line profiles are marked on the EDS maps with a white line. The arrow marks the direction of the line profile. (b) The extracted line profiles for the Co intensity. The sharp drop at the interface in each particle of the (ZIF-67)(ZIF-62)(20/80) sample is indicated by a red arrow. The intensity varies much more slowly across the interface in the flux melted glass $(ZIF\text{-}67)_{0.2}(ZIF\text{-}62)_{0.8}$, indicating a diffuse chemical interface.

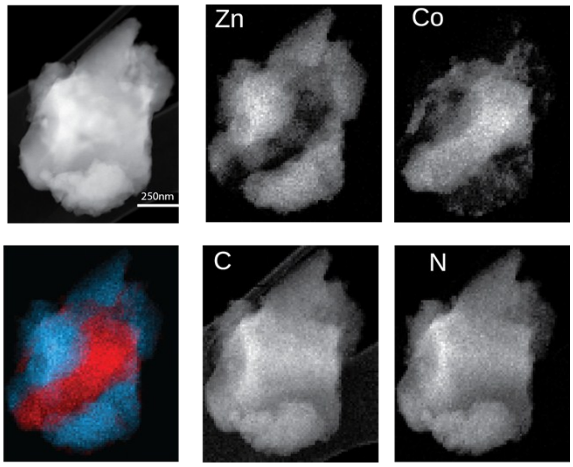

Figure S15. ADF STEM images of (ZIF-67)(ZIF-62)(20/80). Also shown are corresponding EDS elemental maps of C, N, Zn and Co K_{α} signals of (ZIF-67)(ZIF-62)(20/80).

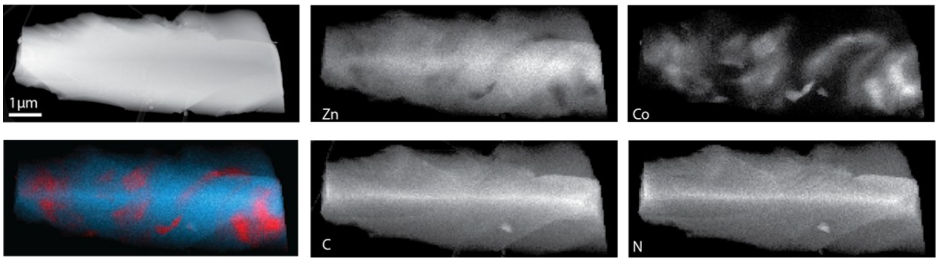

Figure S16. ADF STEM images of a_g(ZIF-67)$_{0.2}$(ZIF-62)$_{0.8}$. Also shown are corresponding EDS elemental maps of C, N, Zn and Co K_{α} signals of a_g(ZIF-67)$_{0.2}$(ZIF-62)$_{0.8}$.

2 Supplementary gas adsorption isotherms and analysis

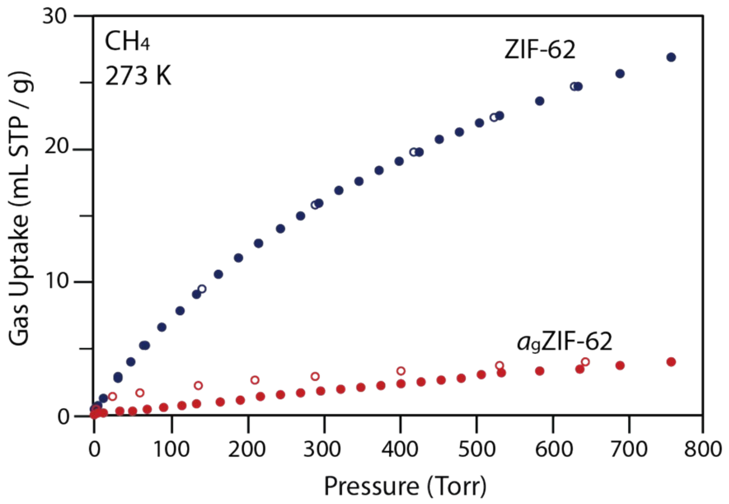

Figure S17. CH_4 isotherms at 273 K for ZIF-62 and a_gZIF-62. Closed symbols represent adsorption. Open symbols represent desorption.

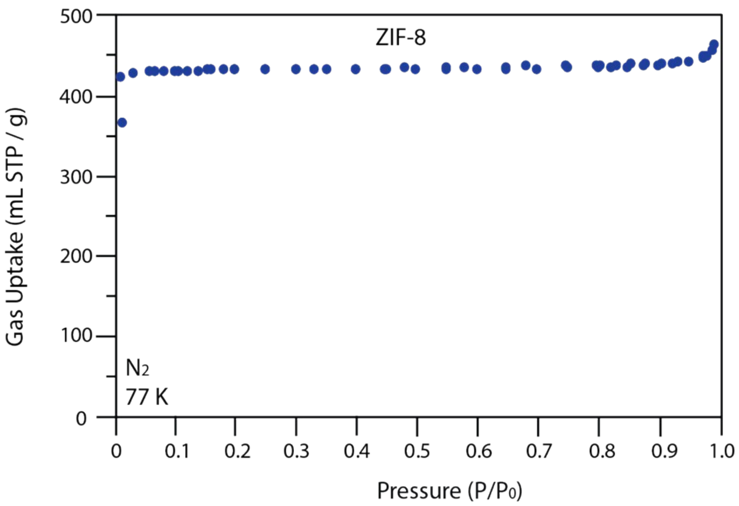

Figure S18. N_2 isotherm at 77 K for ZIF-8.

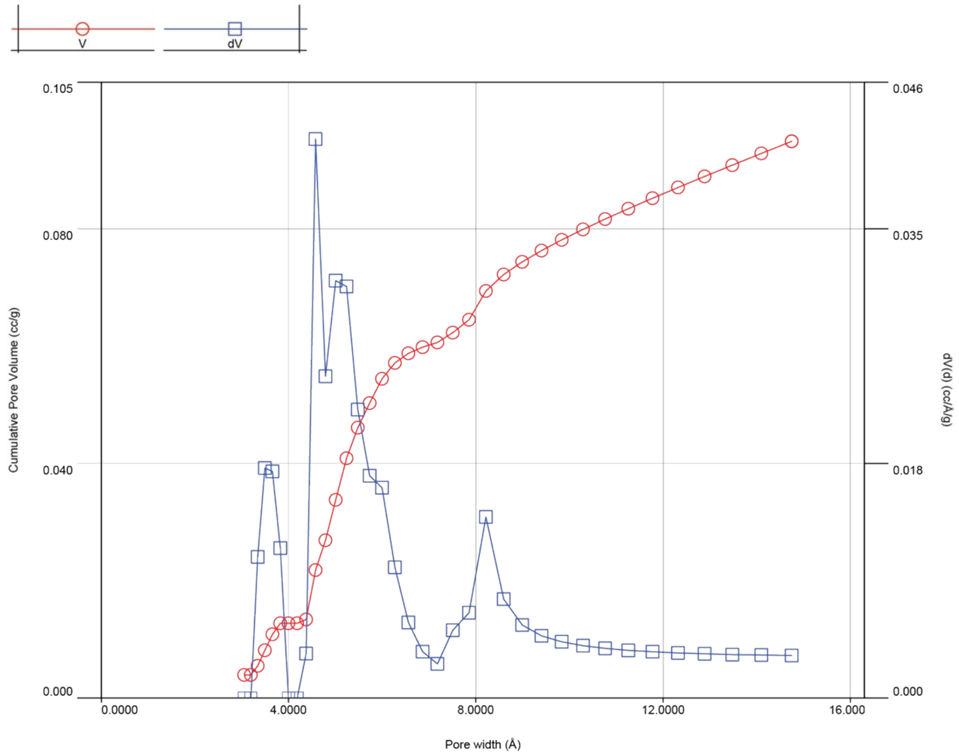

Figure S19. Pore volume distribution of (ZIF-8)(ZIF-62)(20/80), determined from the CO_2 isotherms at 273 K.

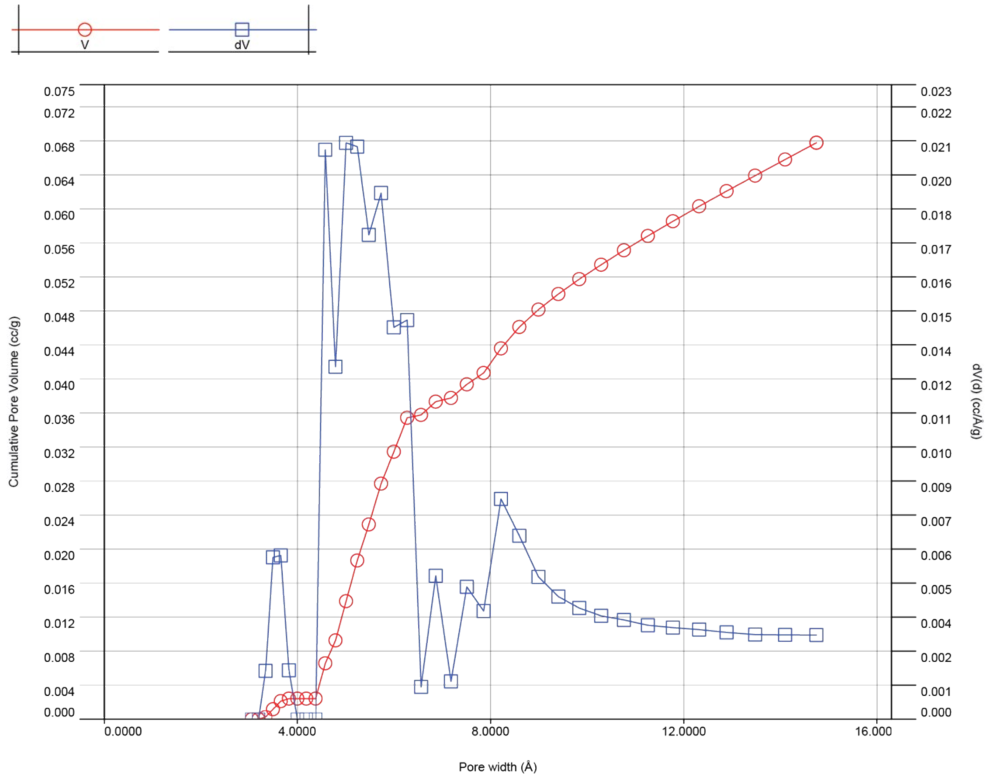

Figure S20. Pore volume distribution of a_g(ZIF-8)$_{0.2}$(ZIF-62)$_{0.8}$ determined from the CO_2 isotherms at 273 K.

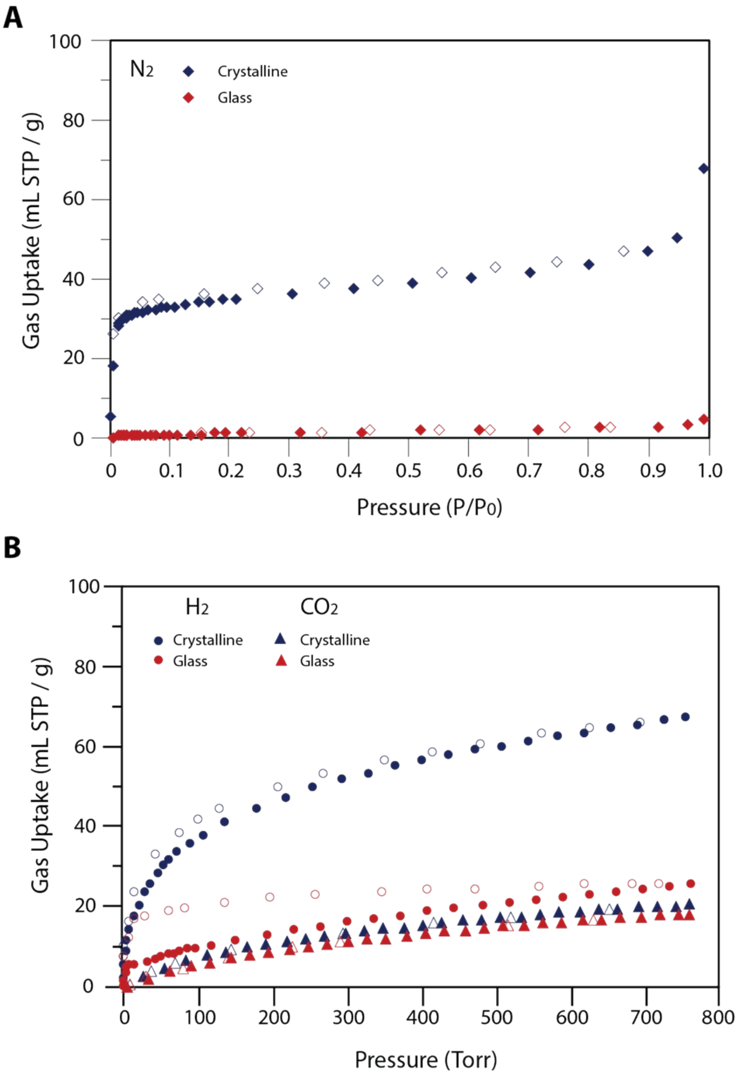

Figure S21. Gas isotherms for (ZIF-67)(ZIF-62)(20/80) and a_g(ZIF-67)$_{0.2}$(ZIF-62)$_{0.8}$. (a) N_2 isotherm at 77 K, (b) CO_2 and H_2 isotherms at 273 K and 77 K respectively.

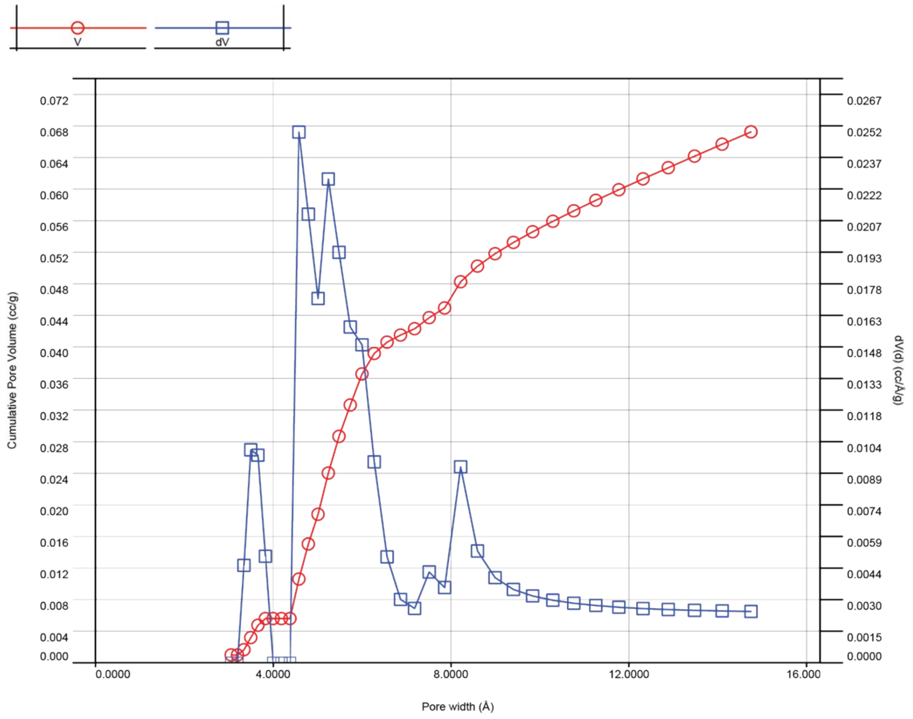

Figure S22. Pore volume distribution of (ZIF-67)(ZIF-62)(20/80), determined from the CO_2 isotherms at 273 K.

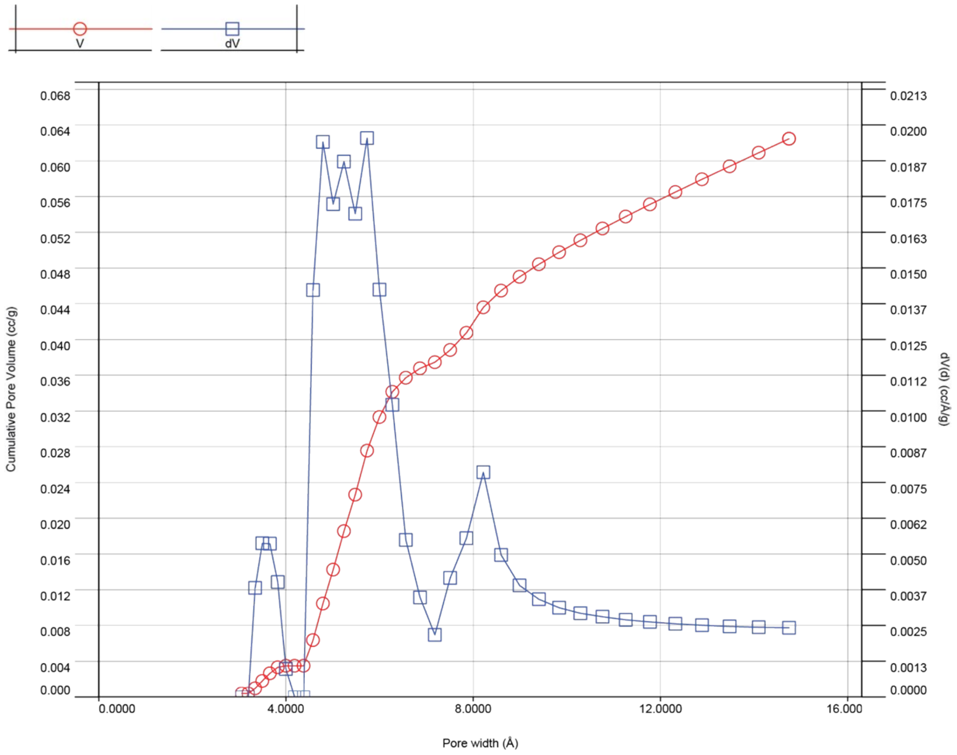

Figure S23. Pore volume distribution of a_g(ZIF-67)$_{0.2}$(ZIF-62)$_{0.8}$, determined from the CO_2 isotherms at 273 K.

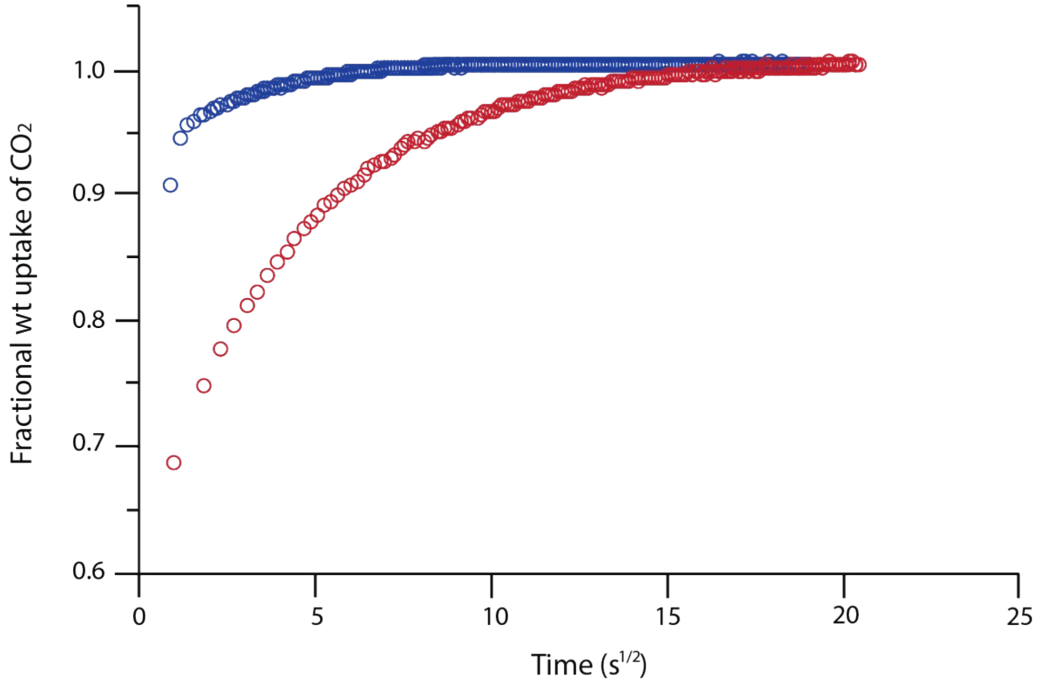

Figure S24. Kinetics of CO_2 adsorption in (blue) (ZIF-67)(ZIF-62)(20/80) and (red) a_g(ZIF-67)$_{0.2}$(ZIF-62)$_{0.8}$.

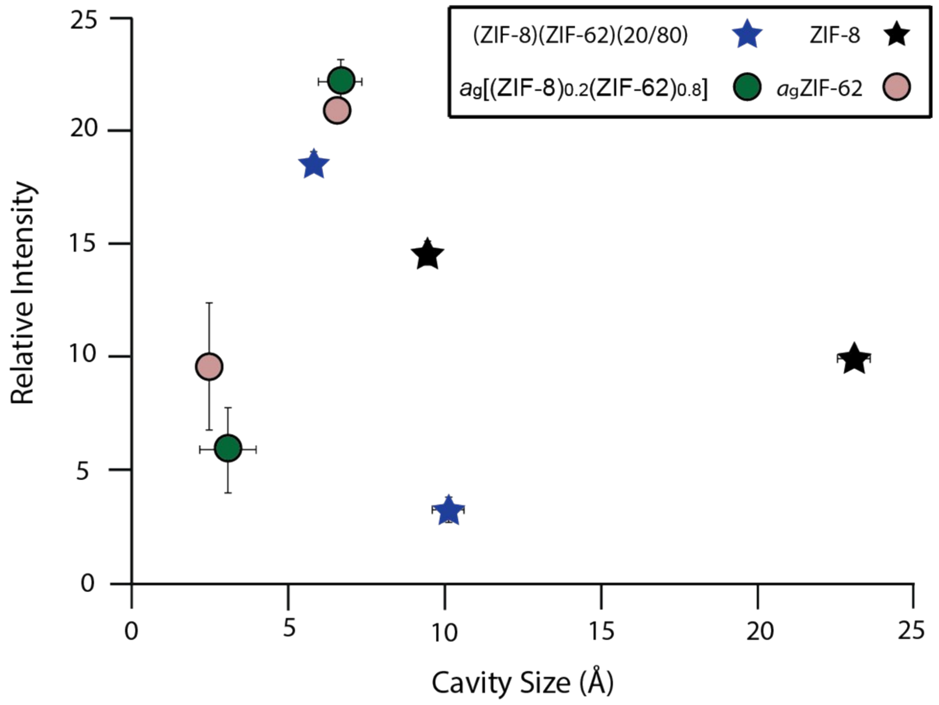

Figure S25. Graphical representation of the cavity size distribution. Determined from the relative intensity of PALS plotted against the average cavity sizes. Data for ZIF-62 glass (a_gZIF-62) taken from a previous publication.[4] Some error bars are smaller than marker width.

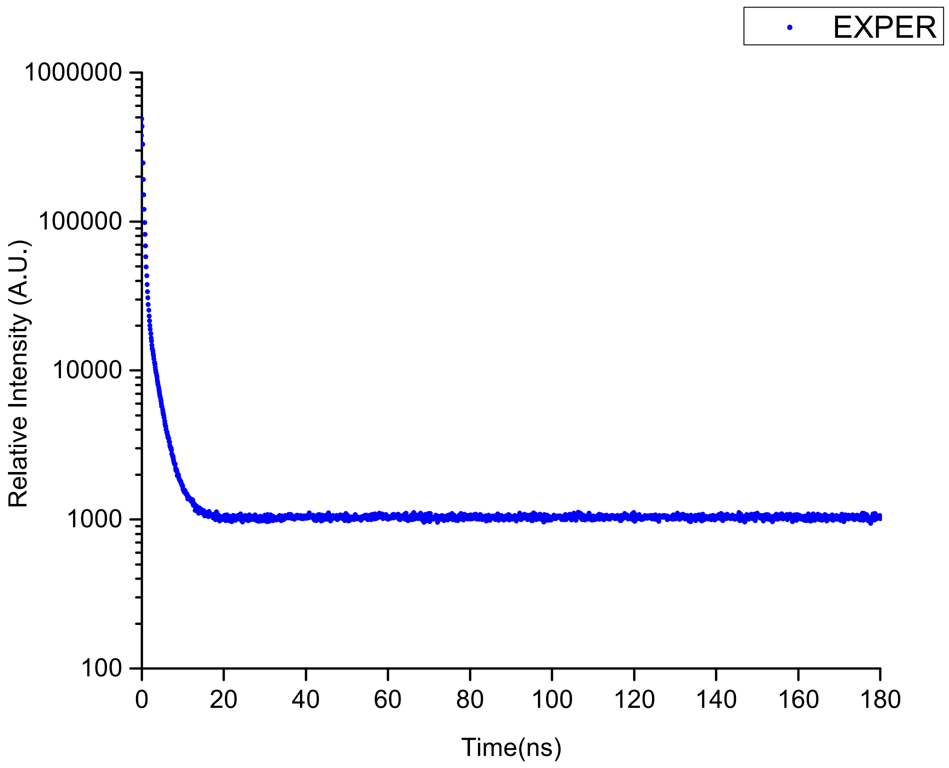

Figure S26. Raw PALS Spectra for the a_g[(ZIF-8)$_{0.2}$(ZIF-62)$_{0.8}$] sample.

3 Simulated gas adsorption isotherms

Crystal structures of ZIF-8 (OFERUN) and ZIF-62 (GIZJOP) were taken from the DFT-optimized Computation-Ready Experimental MOF (CoRE-MOF) database.[5] In the Cambridge Crystallographic Data Center (CCDC)[6], ZIF-62 was deposited with three independent imidazolate linkers and one imidazole ligand disordered between imidazolate (62.5%) and benzimidazolate (37.5%). The imidazolate and benzimidazolate linkers in ZIF-62 were modelled as configuration I and configuration II with partial occupancies of 62.5% and 37.5%, respectively. The structure of ZIF-62 taken directly from DFT-optimized CoRE-MOF database was used as configuration I. To model configuration II, we manually constructed one benzimidazolate linker and optimized the geometry prior to molecular simulations. The DMol3 module of Materials Studio 8.0[7] was used to optimize the geometry and ESP (ElectroStatic Potential) charges were assigned to configuration II.

Modelling amorphous structures is challenging due to the complexity of constructing accurate models. Following the literature,[8] we used a molecular dynamics (MD) method. Initial configurations of ZIF-62 were melted in the NPT ensemble at 1 bar by heating to 1500 K at a rate of 100 K/ps from

300 K, before quenching to 300 K at a controlled rate. These simulations were performed using the Forcite module of Materials Studio. Dreiding was used for bond stretching, angle bending and dihedral torsions as implemented in Materials Studio. The equations of motion were integrated using the velocity Verlet algorithm with a 1 fs time step. Temperature and pressure were controlled using the Nose–Hoover–Langevin thermostat[9] and Berendsen barostat[10] with a relaxation times of 0.1 ps. The van der Waals interactions were computed using an atom-based cutoff distance of 15.5 Å and electrostatic interactions were calculated using the Ewald summation method with an accuracy of 10^{-4} kcal/mol. For quench simulations, MD simulations were performed with a step size of 1 fs up to a total of 1 ns. The lowest energy configurations of ZIF-62 (for both configurations I and II) after these simulations were used as the initial configuration of the adsorption simulations. Structural properties such as accessible pore volume, density, pore limiting diameter (PLD) and the largest cavity diameter (LCD) were calculated using Zeo++ software[11] and listed in Table S3. For pore volume calculations, probe radius was set to zero.

Grand Canonical Monte Carlo (GCMC) simulations were performed as implemented in the RASPA simulation code[12] to compute gas uptakes of CH_4 (273, 298 K), CO_2 (273 K), H_2 (77 K), N_2 (77 K) and O_2 (273 K) in the crystalline samples of ZIF-8 and ZIF-62, and glass sample of ZIF-62. Lennard-Jones (LJ) 12-6 and Coulomb potentials were used to model repulsion-dispersion forces and electrostatic interactions in molecular simulations. The Lorentz-Berthelot mixing rules were used to calculate adsorbent-adsorbate and adsorbate-adsorbate LJ cross interaction parameters. Molecular simulations were performed for 10,000 cycles with the first 5,000 cycles for initialization and the last 5,000 cycles for taking ensemble averages. Three different types of moves including translation, reinsertion and swap of a molecule were considered for spherical molecules. For non-spherical molecules, rotation move was also applied. The cut-off distance was set to 12.8 Å for truncation of the intermolecular interactions and simulation cell lengths were increased to at least 26 Å along each dimension. Periodic boundary conditions were applied in all simulations. The Peng-Robinson equation of state was used to convert the pressure to the corresponding fugacity. All molecular simulations were performed using a rigid framework. More details of these simulations can be found in the literature.[13, 14]

A single-site spherical Lennard-Jones (LJ) 12-6 potential was used to model H_2[15] and CH_4[16] molecules whereas CO_2 was modeled as a three site linear molecule, with three charged LJ interaction sites located at each atom using the EPM2 potential[17]. Similarly, N_2 (O_2) was modeled as a three site molecule with two sites located at two N (O) atoms and the third one located at its center of mass (COM) with partial point charges and the potential parameters of N_2 and O_2 were taken from the literature.[18] The interaction potential parameters for gas molecules were given in Table S4. The atomic charges of glass samples were estimated using the charge equilibration method as implemented in RASPA simulation code. DDEC (density derived electrostatic and chemical) charges were used for crystalline ZIF-8 and ZIF-62 (configuration I) and ESP charges were used for ZIF-62 (configuration II). Ewald

summation method was used to calculate electrostatic interactions. The potential parameters of framework atoms were taken from the literature.[19] These potentials and force fields were selected based on the results of previous studies which showed very good agreement between simulation results and experimentally measured gas uptake data of ZIFs.[19-21]

In order to compare simulation results with the experimentally measured gas uptake data, the absolute gas amount (N_{abs}) obtained directly from GCMC simulations were converted to the excess gas amount (N_{ex}) as follows:

$$N_{ex} = N_{abs} - \rho_g \cdot V_g \quad (1)$$

where ρ_g is the density of adsorbates in gas phase obtained from the Peng-Robinson equation of state and V_g is the pore volume of ZIFs.

Two different configurations of ZIF-62 for both crystalline and glass phases were used to examine gas adsorption. Since the experimental structure of ZIF-62 contains both imidazolate and benzimidazolate linkers together, the average gas adsorption amount in crystalline and glass ZIF-62 was computed using the following equation:

$$N_{ave} = P_1 \cdot n_1 + P_2 \cdot n_2 \quad (2)$$

Here, n_1 (n_2) is the gas uptake computed using configuration I (II), P_1 (P_2) is the probability of configuration I (II), and N_{ave} is the average gas uptake.

To predict the adsorbed gas amount in both ZIF-ZIF adsorbents and ZIF-glassy ZIF adsorbents, we used the following equation:

$$N_{ave\,(\text{ZIF-ZIF})} = \phi_{\text{ZIF-8}} \cdot n_{\text{ZIF-8}} + \phi_{\text{ZIF-62}} \cdot N_{\text{ave-ZIF-62}} \quad (3)$$

$$N_{ave\,(\text{ZIF-glassy ZIF})} = \phi_{\text{ZIF-8}} \cdot n_{\text{ZIF-8}} + \phi_{\text{ZIF-62}_{glass}} \cdot N_{\text{ave-ZIF-62}_{glass}} \quad (4)$$

where, ϕ is the volume fraction, $N_{ave\,(\text{ZIF-ZIF})}$ and $N_{ave\,(\text{ZIF-glassy ZIF})}$ are the predicted adsorbed gas amounts in crystalline mixtures and glasses, respectively.

Supplementary Table 1: Gas adsorption properties for the cobalt-based crystalline and glass samples. Units are mL STP/g.

Gas	**H_2**	**CO_2**	**O_2**	**N_2**	**CH_4**
Temperature / K	**77**	**273**	**273**	**77**	**273**
Kinetic Diameter / Å	2.9	3.3	3.46	3.64	3.76
(ZIF-67)(ZIF-62)(20/80)	66.8	20.8	2.4	67.5	7.5
a_g(ZIF-67)$_{0.2}$(ZIF-62)$_{0.8}$	*25.6*	*18.5*	*2.1*	*4.7*	*5.1*

Supplementary Table 2: Positron annihilation lifetime spectroscopy data. [a] Note due to the long lifetime, RTE is used for ZIF-8 tau4 pore size calculation. All others use TE. [b] Data taken from previous publication.[4] [c] Data taken from previous publication.[22]

	Intensity		Lifetime		Average Pore Diameter (Å)	
Sample	I3 (%)	I4 (%)	τ3 (ns)	τ4 (ns)	D3	D4
ZIF-8[a]	14.6 ± 0.5	9.9 ± 0.2	4.98 ± 0.16	25.43 ± 0.96	9.5 ± 0.2	23.1 ± 0.5
ZIF -4 [c]	6.2 ± 0.6	33.8 ± 1.2	0.98 ± 0.12	2.28 ± 0.01	3.3 ± 0.4	6.2 ± 0.2
(ZIF-8)(ZIF-62)(20/80)	18.6 ± 0.4	3.2 ± 0.6	2.07 ± 0.05	5.73 ± 0.60	5.8 ± 0.1	10.1 ± 0.5
a_gZIF-62[b]	9.6 ± 2.8	20.9 ± 0.3	0.77 ± 0.08	2.55 ± 0.01	2.5 ± 0.4	6.6 ± 0.1
a_g(ZIF-8)$_{0.2}$(ZIF-62)$_{0.8}$	5.9 ± 1.9	22.2 ± 1.0	0.93 ± 0.24	2.61 ± 0.05	3.1 ± 0.9	6.7 ± 0.7

Supplementary Table 3: Structural properties of crystalline and glass samples.

Structures	PLD (Å)	LCD (Å)	Density (g/cm^3)	Pore volume (cm^3/g)
ZIF-8	3.45	11.53	0.90	0.70
ZIF-62 (configuration I)	2.93	5.32	1.14	0.51
ZIF-62 (configuration II)	1.54	4.63	1.36	0.36
a_gZIF-62 (configuration I)	1.64	3.45	1.56	0.25
a_gZIF-62 (configuration II)	2.54	4.75	1.30	0.39

Supplementary Table 4: Interaction potential parameters used for gas molecules.

Molecule	Atom	σ (Å)	ε/k_B (K)	q(e)
H_2	H_2	2.96	34.20	-
CH_4	CH_4	3.73	148.00	-
CO_2	C	2.80	27.02	0.700
	O	3.05	79.01	-0.350
N_2	N	3.306	38.298	-0.405
	COM	0.00	0.00	0.810
O_2	O	3.04	53.02	-0.112
	COM	0.00	0.00	0.224

Supplementary Table 5: Simulated, and experimental N_2 gas adsorption at 195 K and 273 K, for crystalline and glass samples. Units are mL STP/g.

**Temperature / K**	**195**	**273**
(ZIF-67)(ZIF-62)(20/80)	23.0	104.3
Simulated	24.6	207.2
$(ZIF\text{-}8)_{0.2}(ZIF\text{-}62)_{0.8}$	-	1.2
Simulated	-	1.0

4 Supplementary references

1. K. S. Park, Z. Ni, A. P. Cote, J. Y. Choi, R. D. Huang, F. J. Uribe-Romo, H. K. Chae, M. O'Keeffe and O. M. Yaghi, *P Natl Acad Sci USA*, 2006, **103**, 10186-10191.
2. R. Banerjee, A. Phan, B. Wang, C. Knobler, H. Furukawa, M. O'Keeffe and O. M. Yaghi, *Science*, 2008, **319**, 939-943.
3. T. D. Bennett, Y. Z. Yue, P. Li, A. Qiao, H. Tao, G. N. Greaves, T. Richards, G. I. Lampronti, S. A. T. Redfern, F. Blanc, O. K. Farha, J. T. Hupp, A. K. Cheetham and D. A. Keen, *J Am Chem Soc*, 2016, **138**, 3484-3492.
4. A. Qiao, T. D. Bennett, H. T. Tao, A. Krajnc, G. Mali, C. M. Doherty, A. W. Thornton, J. C. Mauro, G. N. Greaves and Y. Z. Yue, *Sci Adv*, 2018, **4**, eaao6827.
5. D. Nazarian, J. S. Camp, Y. G. Chung, R. Q. Snurr and D. S. Sholl, *Chem. Mater.*, 2017, **29**, 2521-2528.
6. F. H. Allen, *Acta Crystallogr., Sect. B: Struct. Sci.*, 2002, **58**, 380-388.
7. *Journal*.
8. C. D. Williams, K. P. Travis, N. A. Burton and J. H. Harding, *Microporous Mesoporous Mater.*, 2016, **228**, 215-223.
9. D. Frenkel and B. Smit, *Understanding Molecular Simulation: From Algorithms to Applications. Second Edition*, Academic Press, London, U.K., 2002.
10. H. J. Berendsen, J. v. Postma, W. F. van Gunsteren, A. DiNola and J. Haak, *J. Chem. Phys.*, 1984, **81**, 3684-3690.
11. T. F. Willems, C. H. Rycroft, M. Kazi, J. C. Meza and M. Haranczyk, *Microporous and Mesoporous Mater.*, 2012, **149**, 134–141.
12. D. Dubbeldam, S. Calero, D. E. Ellis and R. Q. Snurr, *Mol. Simul.*, 2016, **42**, 81–101.
13. D. Dubbeldam, A. Torres-Knoop and K. S. Walton, *Mol. Simul.*, 2013, **39**, 1253–1292.
14. D. Frenkel and B. Smit, *Understanding Molecular Simulation: From Algorithms to Applications*, Academic Press, San Diego, 2nd edn., 2002.
15. V. Buch, *J. Chem. Phys.*, 1994, **100**, 7610–7629.
16. M. G. Martin and J. I. Siepmann, *J. Phys. Chem. B*, 1998, **102**, 2569–2577.
17. J. J. Potoff and J. I. Siepmann, *AIChE J.*, 2001, **47**, 1676-1682.

18. S. Calero, A. Martín-Calvo, S. Hamad and E. García-Pérez, *Chem. Commun.*, 2011, **47**, 508-510.
19. J. Pérez ‐ Pellitero, H. Amrouche, F. R. Siperstein, G. Pirngruber, C. Nieto ‐ Draghi, G. Chaplais, A. Simon ‐ Masseron, D. Bazer ‐ Bachi, D. Peralta and N. Bats, *Chem. - Eur. J.*, 2010, **16**, 1560-1571.
20. W. Morris, B. Leung, H. Furukawa, O. K. Yaghi, N. He, H. Hayashi, Y. Houndonougbo, M. Asta, B. B. Laird and O. M. Yaghi, *J. Am. Chem. Soc.*, 2010, **132**, 11006-11008.
21. J. Liu, S. Keskin, D. S. Sholl and J. K. Johnson, *J. Phys. Chem. C*, 2011, **115**, 12560-12566.
22. A. W. Thornton, K. E. Jelfs, K. Konstas, C. Doherty, A. J. Hill, A. K. Cheetham and T. D. Bennett, *Chem Commun*, 2016, **52**, 3750-3753.
